# Therapeutic targeting of ATR in alveolar rhabdomyosarcoma

**DOI:** 10.1038/s41467-022-32023-7

**Published:** 2022-07-25

**Authors:** Heathcliff Dorado García, Fabian Pusch, Yi Bei, Jennifer von Stebut, Glorymar Ibáñez, Kristina Guillan, Koshi Imami, Dennis Gürgen, Jana Rolff, Konstantin Helmsauer, Stephanie Meyer-Liesener, Natalie Timme, Victor Bardinet, Rocío Chamorro González, Ian C. MacArthur, Celine Y. Chen, Joachim Schulz, Antje M. Wengner, Christian Furth, Birgit Lala, Angelika Eggert, Georg Seifert, Patrick Hundsoerfer, Marieluise Kirchner, Philipp Mertins, Matthias Selbach, Andrej Lissat, Frank Dubois, David Horst, Johannes H. Schulte, Simone Spuler, Daoqi You, Filemon Dela Cruz, Andrew L. Kung, Kerstin Haase, Michela DiVirgilio, Monika Scheer, Michael V. Ortiz, Anton G. Henssen

**Affiliations:** 1 Experimental and Clinical Research Center (ECRC) of the MDC and Charité Berlin, Berlin, Germany; 2 Department of Pediatric Oncology and Hematology, Charité – Universitätsmedizin Berlin, corporate member of Freie Universität Berlin, Humboldt-Universität zu Berlin, Berlin, Germany; 3 Max Delbrück Center for Molecular Medicine, Berlin, Germany; 4 Department of Pediatrics, Memorial Sloan Kettering Cancer Center, New York City, NY USA; 5 Experimental Pharmacology and Oncology (EPO), Berlin, Germany; 6 Charité - Universitätsmedizin Berlin, Corporate Member of Freie Universität Berlin and Humboldt-Universität zu Berlin, Experimental and Clinical Research Center, Charité Campus Buch, Lindenberger Weg 80, 13125 Berlin, Germany; 7 Bayer AG, Berlin, Germany; 8 Berlin Institute of Health, 10178 Berlin, Germany; 9 Institute of pathology, Charité – Universitätsmedizin Berlin, corporate member of Freie Universität Berlin, Humboldt-Universität zu Berlin, Berlin, Germany; 10 Berlin Institute of Health at Charité – Universitätsmedizin Berlin, BIH Biomedical Innovation Academy, BIH Charité Junior Clinician Scientist Program, Charitéplatz 1, 10117 Berlin, Germany; 11 Department of chemistry, biochemistry and pharmacy, Free University of Berlin, Berlin, Germany; 12 German Cancer Consortium (DKTK), partner site Berlin, and German Cancer Research Center (DKFZ), Heidelberg, Germany

**Keywords:** Paediatric cancer, Sarcoma

## Abstract

Despite advances in multi-modal treatment approaches, clinical outcomes of patients suffering from PAX3-FOXO1 fusion oncogene-expressing alveolar rhabdomyosarcoma (ARMS) remain dismal. Here we show that PAX3-FOXO1-expressing ARMS cells are sensitive to pharmacological ataxia telangiectasia and Rad3 related protein (ATR) inhibition. Expression of PAX3-FOXO1 in muscle progenitor cells is not only sufficient to increase sensitivity to ATR inhibition, but PAX3-FOXO1-expressing rhabdomyosarcoma cells also exhibit increased sensitivity to structurally diverse inhibitors of ATR. Mechanistically, ATR inhibition leads to replication stress exacerbation, decreased BRCA1 phosphorylation and reduced homologous recombination-mediated DNA repair pathway activity. Consequently, ATR inhibitor treatment increases sensitivity of ARMS cells to PARP1 inhibition in vitro, and combined treatment with ATR and PARP1 inhibitors induces complete regression of primary patient-derived ARMS xenografts in vivo. Lastly, a genome-wide CRISPR activation screen (CRISPRa) in combination with transcriptional analyses of ATR inhibitor resistant ARMS cells identifies the RAS-MAPK pathway and its targets, the *FOS* gene family, as inducers of resistance to ATR inhibition. Our findings provide a rationale for upcoming biomarker-driven clinical trials of ATR inhibitors in patients suffering from ARMS.

## Abstract (web summary)

Alveolar rhabdomyosarcoma is a clinically challenging disease due to the lack of druggable targets. Here the authors show preclinical evidence for ATR inhibitors as a therapeutic option for alveolar rhabdomyosarcoma.

## Introduction

Rhabdomyosarcomas are the most common soft tissue tumors in childhood^1^. About 25% of cases present histologically as alveolar rhabdomyosarcoma (ARMS) and harbor pathognomonic chromosomal translocations involving genes encoding for the *PAX3* (and less frequently, *PAX7*) and *FOXO1* transcription factors^2,3^. PAX3/7-FOXO1 expression is not only sufficient to drive tumorigenesis^4^, but it is significantly associated with adverse clinical outcome. Rhabdomyosarcomas expressing PAX3/7-FOXO1 have a high metastatic potential and are often refractory to chemotherapy^5^. Despite recent advances in cancer drug development, no new targeted treatment options were clinically approved for metastatic or recurrent rhabdomyosarcomas in the last ~30 years^6^. It is widely accepted that current treatment strategies have reached their limits. PAX3/7-FOXO1-driven rhabdomyosarcomas are rarely associated with therapeutically actionable genetic aberrations^7^. Thus, the identification of new therapeutic strategies for high-risk PAX3/7-FOXO1-expressing rhabdomyosarcoma remains urgent but challenging.

Most cancers depend on active DNA damage repair, explaining why genotoxic agents are among the most effective chemotherapeutic agents in cancer therapy^8^. The therapeutic window of genotoxic agents, however, is often narrow and considerable long-term sequelae occur in patients treated with such agents. Synthetic lethal cellular dependencies have emerged as tumor-specific vulnerabilities, which provide therapeutic targets offering much broader therapeutic windows^9^. In particular, DNA damage response (DDR) pathway dependencies are being successfully exploited for the development of novel therapies. As a prototypical example, *BRCA1* deficient tumors rely on PARP-mediated base-excision DNA repair (BER), a synthetic lethal relationship that was clinically translated in breast and ovarian cancers among other tumor entities^10,11^. Thus, exploiting DDR pathway dependencies may enable the development of novel therapeutic strategies for rhabdomyosarcomas.

**Fig. 1:**
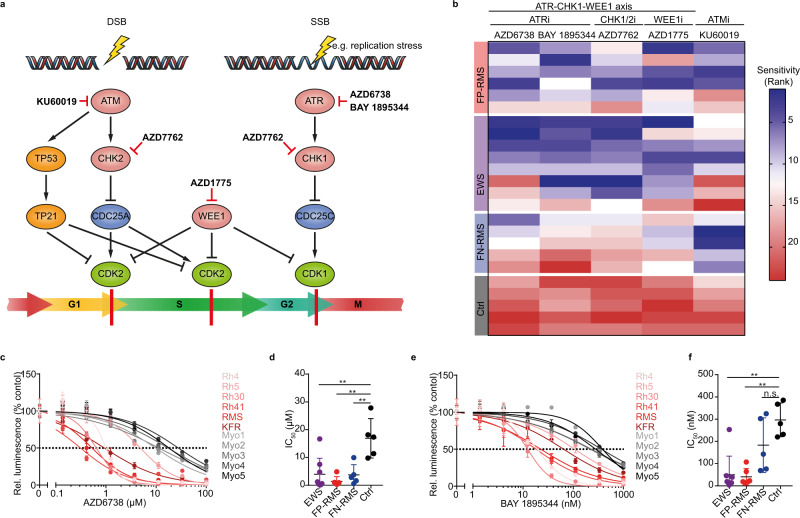
Fusion-positive ARMS cells are sensitive to pharmacological ATR inhibition. **a** Schematic of the DNA damage response pathway and small molecule inhibitor targeting proteins involved. DSB = Double Strand Break, SSB = Single Strand Break. **b** Heatmap showing sensitivity of ARMS (FP-RMS), Ewing sarcoma (EWS), ERMS (FN-RMS), and primary myoblast control cells (Ctrl) to the different DNA damage response inhibitors (blue indicates high sensitivity and red low sensitivity as defined by the rank of IC_50_ values). **c** Dose-response curves of cell viability for FP-RMS cell lines treated with the ATR inhibitor AZD6738 compared to primary myoblasts (*n* = 3). **d** IC_50_ values for FP-RMS, EWS, FN-RMS and Ctrl cells treated with AZD6738 (*P* = 4.10 × 10^−3^; 6.00 × 10^−4^; 6.30 × 10^−3^ for EWS, FP-RMS and FN-RMS vs Ctrl, respectively; from left to right, *n* = 8, 6, 5, and 5 biologically independent cells). **e** Dose-response curves of cell viability for FP-RMS cell lines treated with the ATR inhibitor BAY 1895344 compared to primary myoblasts (*n* = 3). **f** IC_50_ values for FP-RMS, EWS, FN-RMS and Ctrl cells treated with BAY 1895344 (*P* = 2.31 × 10^−4^; 4.59 × 10^−5^; 0.116 for EWS, FP-RMS and FN-RMS vs Ctrl, respectively; from left to right, *n* = 8, 6, 5, and 5 biologically independent cells). All statistical analyses correspond to two-sided student’s t-test; data presented as mean value ± error bars representing standard deviation.

Oncogenes, particularly those encoding for transcription factors and fusion transcription factors, can interfere with the normal function of the DNA replication machinery through deregulation of transcriptional activity^12^. Resulting transcription-induced replication fork stalling leads to activation of DDR pathways, during that unprotected single stranded DNA is bound by Replication Protein A (RPA32), subsequently recruiting the ataxia telangiectasia and Rad3 related (ATR) kinase^13–16^. This process has been termed oncogene-induced replication stress. Upon recognition of the DNA break, ATR activates checkpoint kinase 1 (CHK1) among other factors to stop cells from cycling and to coordinate DNA repair^17^ (Fig. 1a). Unsurprisingly, many tumors depend on ATR activity to proliferate in the presence of oncogene-induced replication stress. Based on this observation, ATR has become a candidate target for pharmacological inhibition in cancer therapy and ATR inhibitors are being tested clinically (eg. NCT03682289, NCT05071209). Considering that molecular features creating synthetic lethal ATR dependencies, including *ATM* and *TP53* loss, MYC proto-oncogene expression, fusion oncogene expression, and PGBD5 expression^18–27^, are present in a subset of rhabdomyosarcoma^7^, we evaluated pharmacological ATR pathway inhibition as a therapeutic option for ARMS. Here, we show that ATR inhibitors exhibit antitumor activity against preclinical models of ARMS and that PAX3-FOXO1 is sufficient to increase sensitivity to ATR inhibition.

## Results

### ARMS cell lines are sensitive to pharmacological ATR pathway inhibition

To identify therapeutically actionable DDR pathway vulnerabilities, we screened six ARMS cell lines, eight Ewing sarcoma cell lines and five embryonal rhabdomyosarcoma (ERMS) cell lines compared to five primary untransformed myoblasts derived from healthy human donors for their sensitivity to small molecule inhibitors of DDR kinases ATR (AZD6738, BAY 1895344^28,29^), ATM (KU60019), CHK1/2 (AZD7762) and WEE1 (AZD1775) (Fig. 1a–f and Supplementary Fig. 1a–p). ARMS cell lines showed varying degrees of sensitivity to small molecule-mediated ATR, ATM, WEE1, and CHK1/2 inhibition, with inhibitory concentrations of 50% reduction in cell viability (IC_50_) ranging between 10 nM and 15 µM (Supplementary Fig. 1a–p). ARMS cells were significantly more sensitive to all inhibitors compared to primary human myoblasts (Fig. 1b–f and Supplementary Fig. 1a–p), suggesting that a therapeutic index exists for these drugs. Sensitivity of ARMS cells to ATR pathway inhibition was similar to that of Ewing sarcoma cell lines (Fig. 1c–f), which were reported to be hypersensitive to ATR inhibition due to fusion oncogene-induced replication stress^30,31^. Thus, ARMS cells are sensitive to pharmacological ATR pathway inhibition.

### ATR inhibition leads to replication stress, genomic instability, apoptosis, and cell cycle disruption in ARMS cells

**Fig. 2:**
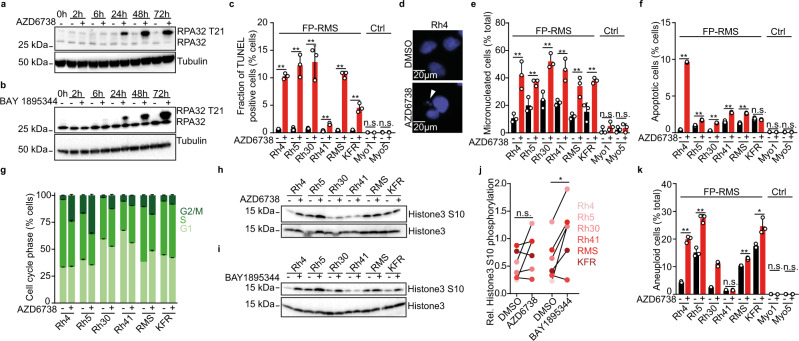
ATR inhibition induces replication stress-associated DNA damage, genomic instability, apoptosis and cell cycle disruption. Western immunoblot of RPA32 phosphorylation at T21 in Rh4 cells treated with ATR inhibitor AZD6738 (750 nM) (**a**) and BAY 1895344 (20 nM) (**b**). **c** Quantification of TUNEL signal in cells treated with AZD6738 for 72 h. (*n* = 3; from left to right, *P* = 5.97 × 10^−6^; 6.51 × 10^−4^; 0.002; 0.001; 6.88 × 10^−6^; 9.04 × 10^−4^; 0.734; 0.980). **d** Representative photomicrographs of micronucleation in cells. White arrow represents micronuclei. **e** Fraction of micronucleated cells after treatment with AZD6738 for 72 h. (*n* = 3, with 50 nuclei counted per replicate; *P* = 0.007; 0.007; 0.004; 0.007; 0.007; 0.004; 0.206; 0.768). **f** Fraction of apoptotic cells after treatment with AZD6738 for 72 h. (*n* = 3; from left to right, *P* = 4.54 × 10^−9^; 7.12 × 10^−4^; 6.12 × 10^−6^; 2.46 × 10^−4^; 6.52 × 10^−5^; 0.313; 0.424; 0.713). **g** Cell cycle phase distribution of cells after treatment with AZD6738 for 72 h. (*n* = 3). Western immunoblot of histone 3 phosphorylation at S10 in six FP-RMS cells treated with AZD6738 (**h**) and BAY 1895344 (**i**) for 2 h. **j** Quantification of changes in histone 3 S10 phosphorylation (*P* = 0.344; 0.016; statistical analysis is sign test). **k** Fraction of aneuploid cells after treatment with AZD6738 for 72 h. (*n* = 3; from left to right, *P* = 2.55 × 10^−5^; 5.45 × 10^−4^; 6.56 × 10^−5^; 0.402; 5.13 × 10^−4^; 0.012; 0.882; 0.565). All statistical analyses correspond to two-sided student’s t-test except for (**j**) data presented as mean value ± error bars representing standard deviation.

Activated ATR is a key mediator of a multifaceted response to DNA replication stress, arrests the cell cycle, blocks replication, and increases repair of stalled replication forks^32,33^. Indeed, short hairpin RNA (shRNA)-mediated knock down of ATR in ARMS cells led to replication stress as evidenced by increased RPA32 T21 phosphorylation (Supplementary Fig. 2a). In line with on-target activity, pharmacological ATR inhibition with both AZD6738 and BAY 1895344, was also accompanied with increased RPA32 T21 phosphorylation (Fig. 2a, b). Consistent with increased replication stress, ARMS cells showed significant accumulation of unrepaired DNA double stranded breaks after incubation with ATR inhibitors or shRNA-mediated ATR knockdown, as measured by terminal deoxynucleotidyl transferase dUTP nick end labeling (TUNEL; Fig. 2c and Supplementary Figs. 2b and 3a). This was accompanied by an increase in micronucleated cells (Fig. 2d, e and Supplementary Fig. 2c), which are typically observed in the context of replication stress^34,35^. Furthermore, cell death, as measured by caspase 3 cleavage, increased in ARMS cells incubated in the presence of an ATR inhibitor or after shRNA-mediated ATR knockdown (Fig. 2f and Supplementary Figs. 2d and 3b). Because of the pivotal role of ATR in controlling the S phase and G2 to M transition checkpoints (Fig. 1a), we measured the cell cycle profiles of cells in response to ATR inhibition, by co-staining cells with 5-Ethynyl-2′-deoxyuridine (EdU) and propidium iodide (PI). After incubation with ATR inhibitors, ARMS cells accumulated in G2/M-phases with a corresponding reduction of cells in S-phase, indicating a bypass of intra-S phase cell cycle checkpoint (Fig. 2g and Supplementary Fig. 3c). This was associated with an increase in histone 3 S10 phosphorylation (Fig. 2h–j and Supplementary Fig. 2e), a marker of mitotic cells^36^, suggesting accumulation in mitosis. The fraction of aneuploid cells was significantly larger after ATR inhibition (Fig. 2k and Supplementary Fig. 3c), pointing at chromosome missegregation due to erroneous repair of unresolved replication intermediates or mitotic catastrophe. Our data suggests that pharmacologic ATR inhibition exacerbates replication stress in ARMS cells, which enter mitosis with unrepaired DNA damage incompatible with cell survival.

### Small molecule ATR inhibition has on-target effects on ATR kinase activity in ARMS cells in vitro at clinically achievable doses

**Fig. 3:**
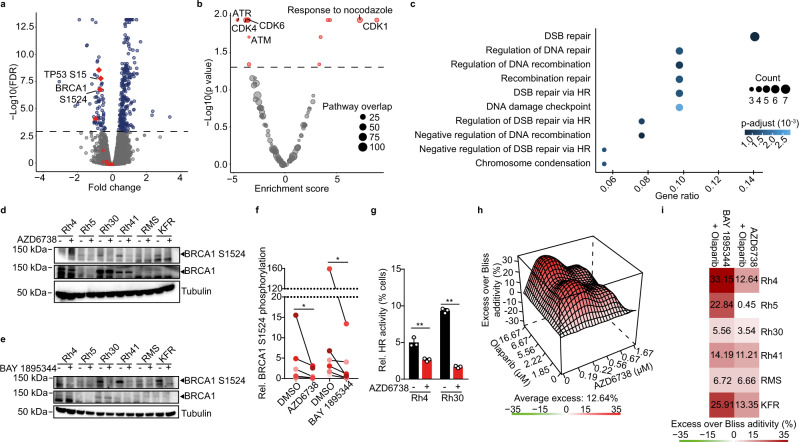
Pharmacological ATR inhibition has on-target activity and leads to reduced BRCA1 activation and repressed homologous recombination. **a** Volcano plot showing relative changes in phospho-peptide abundance in PAX3-FOXO1-expressing Rh30 cells after 2 h of incubation with AZD6738 (750 nM) measured using LC-MS/MS proteomics (red, known ATR targets; dotted line indicating a false discovery rate (FDR) of 0.001). **b** Volcano plot showing relative enrichment of molecular pathways in which differential phospho-peptide abundance was observed in cells treated with AZD6738 (750 nM) compared to DMSO-treated cells (dotted line indicating a false discovery rate of 0.05). **c** Cellular processes significantly enriched in differentially abundant phospho-peptides. Western immunoblotting of BRCA1 S1524 and total BRCA1 in six FP-RMS cells after 2 hours of treatment with AZD6738 (750 nM) (**d**) or BAY 1895344 (20 nM) (**e**). **f** Quantification of changes in BRCA1 S1524 phosphorylation (*P* = 0.016; 0.016 for **d** and **e**, respectively; statistical analysis is sign test). **g** Relative HR activity in Rh4 and Rh30 cells after incubation with AZD6738 (750 nM), measured as GFP reconstitution based on repair of an SceI-mediated DNA lesion via homologous recombination. (*n* = 3 biologically independent experiments; *P* = 0.003; 2.61 × 10^−6^ for Rh4 and Rh30, respectively). **h** Excess over Bliss analysis of combined treatment with olaparib and AZD6738 in Rh4 cells (*n* = 3). **i** Bliss synergy scores for six FP-RMS cell lines treated with AZD6738 and olaparib. All statistical analyses correspond to two-sided student’s t-test except for 3f; data presented as mean value ± error bars representing standard deviation.

We next sought to verify on-target activity of ATR inhibitors on ATR kinase activity as a mechanism of the observed cell cycle disruption, replication stress exacerbation and genomic instability in ARMS cells. To do so, we measured proteome-wide changes in phosphorylation using stable isotope labeling with amino acids in cell culture (SILAC) followed by liquid chromatography with tandem mass spectrometry (LC-MS/MS) phospho-proteomic analysis of cells incubated in the presence of AZD6738. Short-term incubation of ARMS cells with the ATR inhibitor at the same concentrations used in cell assays (Fig. 2a–k) significantly reduced phosphorylation of known ATR kinase target peptides (Fig. 3a), such as the direct ATR target TP53 S15^37^, indicating on-target activity. Using a phosphosite-centered computational analysis tool^38^, we inferred pathway activities after pharmacological ATR inhibition (Fig. 3b). The ATR pathway was the most significantly repressed pathway, again supporting on-target activity of AZD6738 at low doses in ARMS cells. In line with the observed mitotic arrest of cells after ATR inhibition (Fig. 2g–k), peptides from members of the CDK1 pathway and pathways activated in response to nocodazole, an inhibitor of microtubule formation leading to lack of mitotic spindle and M-phase arrest^39^, were phosphorylated at higher degrees after ATR inhibitor treatment (Fig. 3b). Homologous recombination (HR), DNA damage checkpoint and DNA replication pathway proteins, on the other hand, were the most significantly de-phosphorylated after ATR inhibition (Fig. 3c), supporting our conclusion that the observed increase in genomic instability in cells was due to erroneous repair of unresolved replication intermediates and in line with ATR’s role in these pathways^32,33^.

A particularly high degree of differential phosphorylation was measured in BRCA1 peptides (Fig. 3a). BRCA1 is a known substrate of ATR^40^, and is involved in HR at sites of replication stress^41–43^. A cluster of BRCA1 serine residues, including S1524, can be phosphorylated by ATR and serve as key regulatory sites for BRCA1 activity in DNA damage repair^40,44,45^. Using western immunoblotting, we tracked phosphorylation of one of these residues, BRCA1 S1524, in six different ARMS cell lines after 2 h incubation with AZD6738 and BAY 1895344 (Fig. 3d–f). BRCA1 S1524 phosphorylation was significantly reduced following AZD6738 treatment, confirming LC-MS/MS-based measurements (Fig. 3a). Next, we tested whether ATR inhibition affected HR activity by measuring HR on synthetic plasmids transfected into ARMS cells after incubation with AZD6738. Indeed, HR activity on such plasmids was significantly reduced in cells incubated with AZD6738 (Fig. 3g and Supplementary Fig. 3d). Thus, small molecule ATR inhibition has on target activity and represses BRCA1 activity and HR in ARMS cells.

### Combined inhibition of ATR and PARP1 has synergistic anti-tumor effects in ARMS cells

Based on the known^40,44,46,47^ and observed (Fig. 3a–h) effects of ATR inhibition on BRCA1 phosphorylation and HR pathway activity, we hypothesized that the reduced DNA damage repair via HR may increase cells’ sensitivity to pharmacological poly (ADP-ribose) polymerase 1 (PARP1)-trapping on DNA, similarly as observed in *BRCA1*-deficient cancers^10^. Indeed, shRNA-mediated BRCA1 knock down in ARMS cells with three independent shRNAs led to increased sensitivity to PARP1 inhibition, with IC_50_ for olaparib changing from 90.1 µM for shGFP-expressing cells to 5.01, 7.19 and 6.42 µM for three independent shRNAs targeting BRCA1, respectively (Supplementary Fig. 4a, b). This confirmed that in the absence of BRCA1, rhabdomyosarcoma cells are sensitive to PARP-trapping. Consistently, BRCA1 knock down also sensitized rhabdomyosarcoma cells to ATR inhibition (Supplementary Fig. 4c). We hypothesized that due to its effect on BRCA1 phosphorylation and HR activity, pharmacological ATR inhibition could sensitize rhabdomyosarcoma cells to PARP1 inhibition. Indeed, significant synergy of combined AZD6738 or BAY 1895344 and olaparib treatment was detected by Excess over Bliss analysis in six different ARMS cell lines (Fig. 3h–i and Supplementary Fig. 4d–n). Thus, ATR inhibition sensitizes ARMS cells to PARP inhibitor treatment in vitro.

### PAX3-FOXO1 is sufficient to increase replication stress and sensitivity to pharmacological ATR inhibition

Several factors exist in synthetic lethal relationship with ATR^18–26^. To identify which of the known factors may influence rhabdomyosarcoma cells’ sensitivity to ATR inhibition, we assessed their presence in eleven rhabdomyosarcoma cell lines and their association with ATR inhibitor sensitivity (Supplementary Fig. 5a–j). Even though some cell lines that were highly sensitive to ATR inhibition also presented reduced expression of TP53 and ATM, or high *PGBD5* and *CDC25A* mRNA expression, these associations were not statistically significant (Supplementary Fig. 5a–h). HR repair activity did not correlate with sensitivity to AZD6738 (Supplementary Fig. 5i, j), suggesting that differences in endogenous DNA damage levels rather than reduced repair activity was responsible for the observed differences in response to ATR inhibitors. In line with MYCN’s ability to drive replication stress, high MYCN expression was associated with high ATR inhibitor sensitivity (Supplementary Fig. 5a, b). Ectopic expression of MYCN in untransformed mouse myoblast cells, however, did not increase cells’ sensitivity to ATR inhibitors, suggesting that other factors in MYCN-expressing cells drive ATR inhibitor sensitivity (Supplementary Fig. 6a, b).

**Fig. 4:**
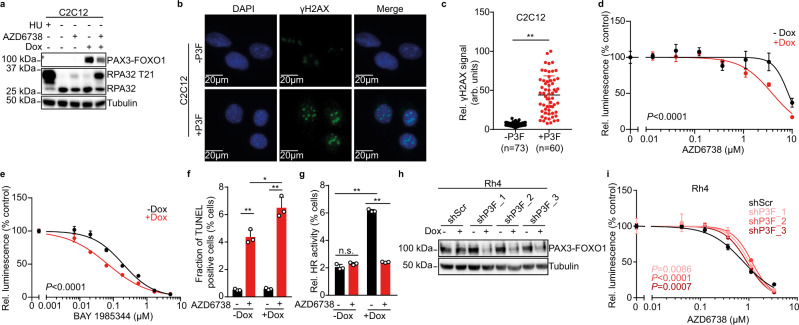
PAX3-FOXO1 is sufficient to increase sensitivity to ATR inhibition in myoblast cells. **a** Western immunoblot of PAX3-FOXO1 and RPA32 phosphorylation at T21 in C2C12 after doxycycline-induced expression of PAX3-FOXO1 (1000 ng/ml for 48 h) and treatment with AZD6738 (750 nM). Hydroxyurea (HU, 1 mM) was used as a control for replication stress. **b** Representative images of H2AX phosphorylation in C2C12 cells after ectopic expression of PAX3-FOXO1. **c** Quantification of H2AX phosphorylation in C2C12 cells after ectopic expression of PAX3-FOXO1 (*P* = 9.57 × 10^−26^). Dose-response curves of cell viability for C2C12 cells after ectopic expression of PAX3-FOXO1 and incubation with AZD6738 (**d**) or BAY 1895344 (**e**) (*n* = 3)**. f** Quantification of TUNEL signal in C2C12 cells after induction of PAX3-FOXO1 with doxycycline and treatment with AZD6738 (*n* = 3; from top to bottom, *P* = 0.016; 1.84 × 10^−4^; 1.99 × 10^−4^). **g** Relative HR activity in C2C12 cells after induction of PAX3-FOXO1 with doxycycline (1000 ng/ml) and incubation with AZD6738 as measured using a GFP reconstitution assay based on repair of an SceI-mediated DNA lesion via homologous recombination (*n* = 3 biologically independent experiments; from top to bottom, *P* = 5.19 × 10^−6^; 3.67 × 10^−7^; 0.114). **h** Western immunoblot of PAX3-FOXO1 in Rh4 cells after doxycycline-induced (1000 ng/mL) expression of shRNAs targeting PAX3-FOXO1 compared to scrambled shRNA control for 48 h. **i** Dose-response curves for Rh4 after doxycycline-induced (1000 ng/mL) expression of shRNAs targeting PAX3-FOXO1 compared to scrambled shRNA control and treated with AZD6738 (*n* = 3). All statistical analyses correspond to two-sided student’s t-test; data presented as mean value ± error bars representing standard deviation.

Based on previous reports showing that chimeric transcription factors, such as EWS-FLI1 in Ewing sarcoma, can themselves render cells sensitive to ATR inhibition through induction of replication stress^30,31^, we hypothesized that PAX3-FOXO1 may contribute to replication stress and sensitivity to ATR inhibition in ARMS. To test this, we ectopically expressed PAX3-FOXO1 in untransformed mouse myoblast cells (C2C12, Fig. 4a). In line with oncogene-induced replication stress, ectopic PAX3-FOXO1 expression was associated with increased phosphorylation of RPA32 at T21, particularly in response to ATR inhibition with AZD6738 (Fig. 4a). This change in RPA32 phosphorylation was also observed in cells treated with hydroxyurea (HU), a potent inducer of replication stress (Fig. 4a). Consistent with increased oncogene-induced replication stress, H2AX phosphorylation, an early marker of DNA damage, increased in cells expressing PAX3-FOXO1 (Fig. 4b, c). This was accompanied by significantly increased sensitivity to the two structurally diverse ATR inhibitors, AZD6738 and BAY 1895344 (Fig. 4d, e). Furthermore, cells expressing PAX3-FOXO1 showed higher levels of TUNEL positive cells in response to AZD6738 than their counterpart control (Fig. 4f). Interestingly, overexpression of PAX3-FOXO1 led to an increase in HR activity, which was repressed by ATR inhibitor treatment (Fig. 4g). This indicates that myoblast cells depend on ATR activity in the presence of PAX3-FOXO1-induced replication stress to maintain increased HR activity.

To test whether PAX3-FOXO1 was required for ATR inhibitor sensitivity, we induced the expression of shRNAs directed against *PAX3-FOXO1* mRNA in PAX3-FOXO1-expressing ARMS cells (Fig. 4h). shRNA-mediated depletion of PAX3-FOXO1 led to reduced cell survival in ARMS cells (Supplementary Fig. 7a), consistent with the essential role of PAX3-FOXO1 in ARMS^48^. Even though the toxicity of PAX3-FOXO1 knockdown may affect the interpretation of our results, a significantly reduced sensitivity to ATR inhibitor treatment was observable after shRNA-mediated PAX3-FOXO1 knockdown (Fig. 4i, Supplementary Fig. 7b). Thus, the pathognomonic fusion oncoprotein PAX3-FOXO1 is not only sufficient to increase replication stress and ATR inhibitor sensitivity in myoblast cells, but is also required for ATR inhibitor sensitivity in ARMS cells.

### A genome wide CRISPR activation screen identifies molecular factors reducing ATR inhibitor sensitivity in ARMS cells

**Fig. 5:**
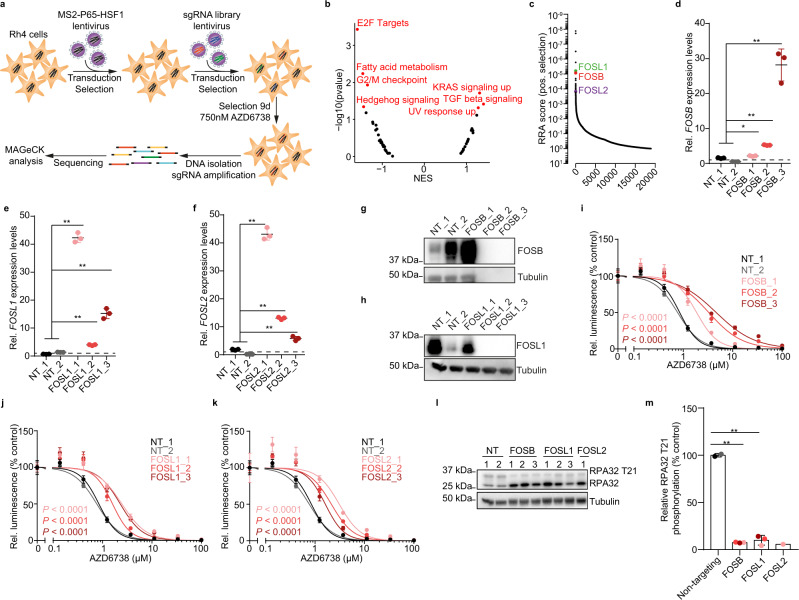
A genome wide CRISPR-based activation screen identifies molecular modifiers of sensitivity to ATR inhibition in PAX3-FOXO1-expressing ARMS cells. **a** Schematic representation of the genome wide CRISPRa screen experimental design. **b** Enrichment score for the GSEA hallmark pathways based on sgRNA enrichment. **c** Waterfall plot showing the positive robust rank aggregation (RRA) score of sgRNAs in Rh4 cells incubated in the presence of AZD6738 for 9 days compared to DMSO treated cells as analyzed using MAGeCK. *FOSB* (**d**, *P* = 0.014; 5.45 × 10^−6^; 1.17 × 10^−6^), *FOSL1* (**e**, *P* = 9.09 × 10^−11^; 5.16 × 10^−6^; 2.19 × 10^−7^) and *FOSL2* (**f** *P* = 9.87 × 10^−10^; 1.49 × 10^−7^; 1.15 × 10^−4^) mRNA expression measured using RT-qPCR in Rh30 cells expressing dCas9, lentiMPH and sgRNAs targeting *FOSB*, *FOSL1* or *FOSL2* (*n* = 3). Western immunoblot of FOSB (**g**) and FOSL1 (**h**) in Rh30 cells stably expressing dCas9, lentiMPH and sgRNAs targeting *FOSB* and *FOSL1*, respectively. Relative cell viability of Rh30 cells stably expressing dCas9, lentiMPH and sgRNAs targeting *FOSB* (**i**), *FOSL1* (**j**) and *FOSL2* (**k**) in the presence of varying concentrations of AZD6738. **l** Western immunoblot of RPA32 phosphorylation at T21 in Rh4 cells expressing sgRNAs targeting FOS family members *FOSB*, *FOSL1* or *FOSL2*. **m** Quantification of RPA32 phosphorylation at T21compared to the corresponding non-targeting control, (*P* = 2.66 × 10^−6^; 1.78 × 10^−4^, respectively). All statistical analyses correspond to two-sided student’s t-test; data presented as mean value ± error bars representing standard deviation.

Successful clinical translation of targeted therapies can be hampered by rapid occurrence of resistance^49^. Therefore, we aimed to identify factors altering sensitivity of ARMS cells to ATR inhibition, even in the presence of PAX3-FOXO1. To identify such factors, we used a genome-wide CRISPR-Cas9-based gene activation screen (CRISPRa) targeting over 70,000 genomic loci covering 20,000 gene promoters^50^. PAX3-FOXO1-expressing cells were genetically engineered to express endonuclease-deficient Cas9 (dCas9), transcriptional activation complex members and transduced with a single guide RNA (sgRNA) library. Next, cells were incubated for 9 days in the presence of the ATR inhibitor AZD6738 (Fig. 5a). sgRNAs significantly depleted in cells exposed to AZD6738 contained known sensitizers to ATR inhibition such as *MYC* and *CDC25A* (Supplementary Fig. 8a)^22^. Consistently, an unsupervised pathway analysis identified E2F targets and G2/M checkpoint genes enriched in sgRNAs depleted after AZD6738 exposure, i.e., associated with increased ATR inhibitor sensitivity (Fig. 5b). sgRNAs with increased abundance after AZD6738 exposure, on the other hand, were significantly enriched for KRAS-activated gene pathway members (Fig. 5b). This suggests that the RAS-MAPK pathway may promote ATR inhibitor resistance.

Interestingly, *FOSB, FOSL1*, and *FOSL2*, members of the AP-1 transcription factors and downstream targets of the RAS-MAPK pathway^51–54^, were amongst the top genes targeted by sgRNAs with increased abundance in the presence of AZD6738 (Fig. 5c and Supplementary Fig. 8b). Efficient induction of *FOSB, FOSL1* and *FOSL2* mRNA and protein expression by CRISPRa was confirmed in two cell lines using RT-qPCR and western immunoblotting, respectively (Fig. 5d–h and Supplementary Fig. 8c–g). In line with increased resistance to ATR inhibition, cells expressing diverse *FOSB, FOSL1* and *FOSL2*-targeting sgRNAs and dCas9 were significantly less sensitive to ATR inhibition compared to cells expressing non-targeting sgRNAs, as evidenced by changes in dose-response relationship of two independent ARMS cell lines (Fig. 5i–k and Supplementary Fig. 8h–j). The AP-1 complex, including the *FOS* gene family members, are known modulators of DDR^55,56^, leading us to hypothesize that *FOSB, FOSL1,* and *FOSL2* expression may reduce baseline replication stress in ARMS cells even in the presence of PAX3-FOXO1. Indeed, CRISPRa-driven *FOS* gene family member expression was sufficient to reduce steady-state RPA32 T21 phosphorylation in ARMS cells, indicating reduced replication stress (Fig. 5l, m). Thus, *FOS* gene family member expression represents a mechanism through which ARMS cells can reduce replication stress, which is accompanied by reduced ATR inhibitor sensitivity.

### ATR inhibitor resistance is associated with increased RAS-MAPK signaling and FOSB expression

**Fig. 6:**
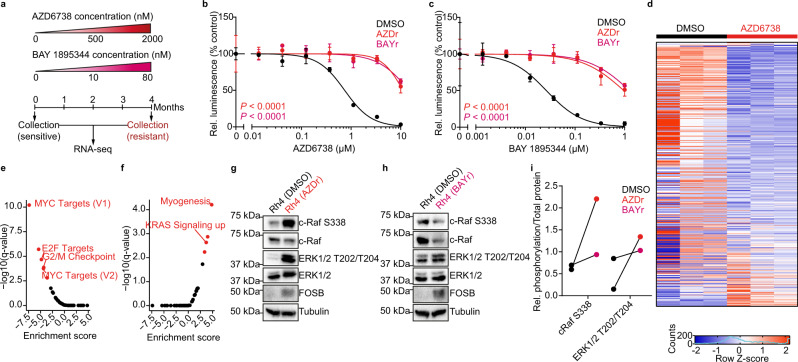
ATR inhibitor-resistant cells express FOSB and activated MAPK pathway. **a** Schematic representation of the generation of ATR inhibitor-resistant cells by long-term exposure to increasing doses of the ATR inhibitors AZD6738 and BAY 1895344. Dose-response curves of cell viability for resistant cells after incubation with AZD6738 (**b**) or BAY 1895344 (**c**) compared to treatment-naïve cells (*n* = 3 biologically independent experiments). **d** Heatmap of the 500 most variable genes based on RNA sequencing. Enrichment score for the GSEA hallmark pathways in ATR inhibitor-resistant cells based on RNA sequencing data, showing negatively (**e**) and positively enriched pathways (**f**). Western immunoblotting of RAS-MAPK pathway members in cells resistant to AZD6738 (**g**) or BAY 1895344 (**h**) compared to treatment-naïve cells. **i** Quantification of changes in c-Raf and ERK1/2 phosphorylation as measured in (g-h). All statistical analyses correspond to two-sided student’s t-test; data presented as mean value ± error bars representing standard deviation.

To further investigate molecular mechanisms impeding ATR inhibitor sensitivity, we generated ATR inhibitor-resistant ARMS cells by incubating cells in the presence of AZD6738 and BAY 1895344 at increasing concentrations over a period of 4 months (Fig. 6a). We confirmed resistance of these cells to both inhibitors through dose-response measurements (Fig. 6b, c). Next, we performed RNA sequencing of ATR inhibitor-resistant cells and control cells and compared their gene expression. ATR inhibitor-resistant cells differed significantly with regards to their gene expression (Fig. 6d and Supplementary Fig. 9a). In line with the results from our CRISPRa screen (Fig. 5), gene expression pathway analysis identified MYC, E2F target, and G2/M checkpoint genes as being repressed in resistant cells (Fig. 6e and Supplementary Fig. 9b–e). The KRAS pathway, on the other hand, was one of the top pathways enriched in genes highly expressed in ATR inhibitor-resistant cells (Fig. 6f and Supplementary Fig. 9f, g). We confirmed higher RAS-MAPK pathway activity in ATR inhibitor-resistant cells compared to non-resistant cells by measuring c-Raf S338 and ERK1/2 T202/T204 phosphorylation (Fig. 6g–i). High RAS-MAPK activity was associated with high FOSB expression (Fig. 6g, h), further strengthening a functional link between RAS-MAPK activity, FOS family member expression, and ATR inhibitor resistance.

### ATR inhibition suppresses tumor growth in ARMS patient-derived xenografts

**Fig. 7:**
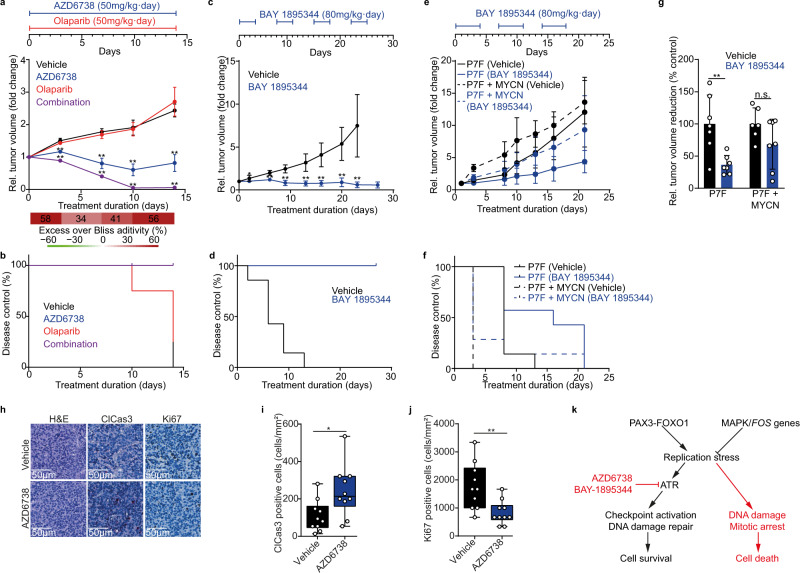
ATR inhibition sensitizes ARMS PDXs to PARP1 inhibition in vivo. **a** Tumor volume change of an ARMS PDX treated with AZD6738, olaparib or both compared to control (*n* = 4 mice per group; bottom, excess over Bliss additivity, ** *P* < 0.01). **b** Kaplan–Meier curve showing tumor doubling time after treatment. **c** Tumor volume change of the ARMS PDX treated with BAY 1895344 as compared to control (*n* = 7 mice per group; top, timeline of the drug schedule, ** *P* < 0.01). **d** Kaplan–Meier curve showing tumor doubling time after treatment. **e** Tumor volume change of an ARMS PDX harboring a PAX7-FOXO1 and a relapse with an additional MYCN amplification, treated with BAY 1895344 as compared to control (*n* = 7 mice per group; top, timeline of the drug schedule). **f** Kaplan–Meier curve showing tumor doubling time after treatment. **g** Tumor volume reduction at the endpoint of the treatment with BAY 1895344 or vehicle in a PAX7-FOXO1 ARMS PDX and a PAX7-FOXO1 MYCN amplified ARMS PDX (*n* = 6 mice for vehicle and *n* = 7 mice for BAY 1895344 treatment; *P* = 0.004; 0.117, respectively.) **h** Representative immunohistochemistry staining for cleaved Caspase3 and Ki67. Quantification of cleaved Caspase3 (**i**) and Ki67 (**j**) (*n* = 10 sections of 275µmx275µm; *P* = 0.005). **k** Schematic of our proposed model. PAX3-FOXO1 induces replication stress, which activates the ATR signaling pathway, promoting checkpoint activation and DNA repair. With ATR inhibitors, replication stress cannot be repaired, leading to DNA damage accumulation, mitotic arrest, and cell death. A proposed counteractive measure is the activation of the RAS-MAPK pathway, in particular *FOS* genes, to reduce replication stress. All statistical analyses correspond to two-sided student’s t-test; data presented as mean value ± error bars representing standard deviation. Box plots (**i** and **j**) show center line as median, box limits as upper and lower quartiles, whiskers as minimum to maximum values.

Based on our results in human cell line models, we next sought to explore the effect of single agent ATR inhibition and its combination with olaparib in mice harboring patient-derived rhabdomyosarcoma xenografts (PDX). We measured the antitumoral effect of ATR inhibitors in an ARMS PDX derived from a 16-year-old female patient presenting with a relapsed ARMS in her forefoot. Histological analysis of the PDX and matching patient tumor confirmed that the PDX model adequately reflected ARMS histologically and expressed PAX3-FOXO1 (Supplementary Fig. 10a). Remarkably, this PDX was resistant to vincristine and ifosfamide, the two standard-of-care agents in ARMS therapy regimens^6^ (Supplementary Fig. 10b–d). Both ATR inhibitors AZD6738 and BAY 1895344 had no significant effects on body weight stability (Supplementary Fig. 10e, f). Only mild reductions in erythrocyte counts were observed over the course of BAY 1895344 treatment (Supplementary Fig. 10g–o), consistent with the known on-target off-tumor toxicity of ATR inhibitors^29,57^. No histopathological differences were observed in six organs in mice treated with BAY 1895344 (Supplementary Fig. 10p), including muscle. In line with our observations in vitro, single-agent AZD6738 or BAY 1895344 treatment led to significant reductions in tumor burden over time in mice harboring the ARMS PDX (Fig. 7a–d). Next, we treated another ARMS PDX derived from a 4-year-old female with a PAX7-FOXO1-expressing relapsed ARMS in her left paraspinal mass. Treatment with BAY 1895344 significantly delayed tumor progression (Fig. 7e–g), suggesting that PAX7-FOXO1-harboring ARMS also respond to pharmacological ATR inhibition. In parallel, we treated a PDX derived from a different relapse of the same patient, in which a de novo *MYCN* amplification was detected. Even though BAY 1895344 treatment was accompanied by reduced PDX growth, the effects were less pronounced compared to the PDX lacking the *MYCN* amplification. Even though we cannot exclude the existence of additional genetic changes between the two PDX models derived from the same patient, the fact that a *MYCN* amplification was not associated with increased sensitivity of the PDX to ATR inhibition indicates that MYCN expression was not sufficient to alter ATR inhibitor sensitivity, in line with our observations in vitro (Supplementary Fig. 6). Tumors from mice treated with AZD6738 showed increased Caspase 3 cleavage and decreased Ki67 staining compared to tumors from mice treated with the vehicle control, suggesting that ATR inhibitor treatment led to increased cell death and decreased cell proliferation (Fig. 7h–j). Addition of olaparib to AZD6738, as currently explored in clinical trials in other tumor entities (NCT03682289), significantly potentiated the anti-tumor effects, leading to full regression of the PDX tumors (Fig. 7a). Loss of mouse weight after 10 days of combined AZD6738 and olaparib treatment, however, indicated increased toxicity compared to single agent treatment (Supplementary Fig. 10f). Thus, ATR inhibition has anti-tumor activity against preclinical ARMS models, which may be clinically translatable.

## Discussion

We have found that preclinical models of ARMS are sensitive to pharmacological ATR inhibition. Consistent with previous reports of other oncogenic fusion genes inducing replication stress (e.g., in Ewing sarcoma^31^), expression of PAX3-FOXO1 was sufficient to increase replication stress, which required both DNA damage repair and DNA damage signaling, resulting in apoptosis if impaired by the selective inhibition of ATR (Fig. 7k). Untransformed mouse myoblast cells engineered to express PAX3-FOXO1, as well as PAX3-FOXO1-expressing rhabdomyosarcoma cell lines, accumulated unrepaired DNA damage and underwent apoptosis upon treatment with selective inhibitors of ATR signaling. These effects, observed particularly in PAX3-FOXO1-expressing rhabdomyosarcoma cells, were associated with on-target effects of ATR inhibition, such as decreased phosphorylation of BRCA1 and homologous recombination activity, and were accompanied by induction of genomic instability, increased mitotic arrest and apoptosis. In turn, single-agent treatment with two different inhibitors of ATR exhibited potent antitumor activity against high-risk patient-derived ARMS models. Moreover, decreased BRCA1 and homologous recombination activity through pharmacological ATR inhibition sensitized cells to PARP1 inhibition. When combined, ATR and PARP1 inhibitors exhibited strong antitumor activity against patient-derived ARMS models resistant to the current standard-of-care treatment.

Human cancers require active DNA damage repair for survival. As a result, selective inhibitors of ATR-mediated DNA damage repair signaling are used to target tumors with intrinsic deficiencies in DNA repair or high abundance of DNA damage^20,24,27,30,31,46,47,58^. Dissecting the molecular mechanisms of susceptibility to ATR inhibitors has been the subject of extensive investigations in the past years^59^. We and others have found inducers of ATR inhibitor susceptibility, such as PGBD5 recombinase activity in embryonal tumors^21^, oncogene-induced replication stress, *ATM* loss, and TP53 deficiency^20,27,60^. Our current work revealed a specific dependency conferred by high steady-state replication stress in alveolar, PAX3-FOXO1-expressing rhabdomyosarcoma. In contrast to previous reports, we did not observe a statistically significant association between these factors and ATR inhibitor sensitivity in ARMS cell lines, suggesting that additional factors influence DDR pathway dependencies in ARMS. In line with the elimusertib phase I/II clinical trial data showing lack of response in 7 out of 11 patients with ATM aberrations, we also did not find *ATM* loss to be associated with increased ATR inhibitor sensitivity in ARMS cells. Pharmacological inhibition of DNA damage signaling kinases exhibited a specific response profile, with ATR- selective inhibitors showing enhanced replication stress-dependent anti-tumor activity. Notably, CHK1, a downstream target of ATR, is inhibited by prexasertib, which is currently being clinically investigated in combination with chemotherapy for patients with relapsed rhabdomyosarcoma (NCT04095221). Given their varied potency and selectivity, it is possible that other selective DNA damage signaling inhibitors can also effectively target replication stress-induced dependencies in rhabdomyosarcoma. Because ATR is also activated by specific DNA structures such DNA–RNA hybrid R-loops, which can be the cause of oncogene-induced DNA replication stress^31^, the preferential activity of ATR inhibitors in PAX3-FOXO1-expressing cells may also be due to the formation of such structures. We provide evidence that PAX3-FOXO1 expression, at least in part, contributes to replication stress and sensitivity to ATR inhibition, which is consistent with previous reports of fusion oncogene-induced replication stress in Ewing sarcoma^30,31^.

*MYCN* has been described as a direct target of PAX3-FOXO1 and is itself a potent inducer of replication stress^61^. High MYCN expression was also detected in PAX3-FOXO1-expressing cells and was positively associated with ATR inhibitor sensitivity, but ectopic MYCN expression did not lead to increased ATR inhibitor sensitivity. Furthermore, ATR inhibitors showed higher antitumor activity in a PDX harboring a PAX7-FOXO1 fusion compared to a PDX from the same patient with a *MYCN* amplification. Thus, MYCN does not seem to contribute to ATR inhibitor sensitivity in ARMS to the same extend as it does in other tumor entities.

ATR is essential for intra-S phase and G2/M checkpoint activation^13,16,25,32^. When checkpoints are constitutively active, cells can undergo checkpoint adaptation to continue proliferating despite the presence of DNA damage^62,63^. We anticipate that susceptibility to ATR inhibitors may also depend on tumor-specific mechanisms of checkpoint adaptation. CHK1 and CDK1 can promote checkpoint adaptation by mediating forced mitotic entry^64,65^. Inhibition of ATR could exacerbate the effect of checkpoint adaptation by suppressing checkpoint activation. Consistently, we observed accumulation of cells in mitosis and increased activation of CDK1 targets in our phosphoproteomic profiling after ATR inhibition. In line with checkpoint adaptation promoting DNA damage accumulation and genomic instability^63^, we observed high degrees of genomic instability in PAX3-FOXO1-expressing cells treated with ATR inhibitors. Intriguingly, PAX3-FOXO1 can itself promote checkpoint adaptation in rhabdomyosarcoma cells through induction of PLK1 expression, which in turn activates CDK1 and forces mitotic entry^66^. It is tempting to speculate that PAX3-FOXO1-induced checkpoint adaptation may also influence ATR inhibitor sensitivity.

Even though results of clinical trials with ATR inhibitors in adults have shown promising single agent antitumor activity in various tumor entities, some patients progress or relapse after some time^57,67^. Thus, identifying molecular mechanisms of ATR inhibitor resistance is of paramount clinical importance, as it may enable the identification of clinical biomarkers that help predict ATR inhibitor susceptibility and can be used to monitor resistance development. Our genome wide CRISPRa screen and models of ATR inhibitor resistance identified the RAS-MAPK pathway and its downstream effectors, the *FOS* family of transcription factors, as modulators of ATR sensitivity. How *FOS* gene family expression leads to reduced steady-state replication stress, is still unresolved (Fig. 7h). A study in osteosarcoma showed that expression of *FOS* protects cells from replication stress by inducing CHK1 and facilitates transformation by RAS-MAPK^68^. Based on our findings and previous reports, it is tempting to speculate that pharmacological RAS-MAPK inhibition may enhance ATR inhibitor sensitivity or delay onset of resistance in ARMS.

In conclusion, we here present preclinical evidence supporting a molecularly targetable therapeutic option for ARMS, for which current treatment options have been exhausted and prognosis remains dismal. Our findings warrant the future investigation of ATR inhibitors in clinical trials, such as the currently undergoing phase I/II trial of BAY 1895344 (Elimusertib) in relapsed PAX3-FOXO1-expressing rhabdomyosarcoma (NCT05071209). We hope that our in-depth analysis of molecular factors influencing ATR inhibitor sensitivity will help guide predictive biomarker development.

## Methods

### Study design

The purpose of this study was to examine the effects of ATR inhibition in preclinical models of rhabdomyosarcoma and identify potential biomarkers to select patients that could benefit from small molecule ATR inhibitor treatment. We first determined the inhibitory activity of the ATR inhibitors in rhabdomyosarcoma cell models, and compared these cells based on known determinants of ATR inhibition sensitivity, as well as PAX3-FOXO1, a molecular feature of ARMS. We analyzed the effects of AZD6738 treatment on genomic instability (including double strand break formation, micronucleation, and apoptosis) and on protein phosphorylation. This study was performed following the guidelines recommended by Carola A.S. Arndt for childhood and adolescent tumors, namely five to eight cell lines per disease, for which we validated the expression of the target gene, included 72 h IC_50_ determination to each drug, and explored potential two-drug combinations^69^. Outliers were not excluded unless technical errors were present. For the CRISPRa screen, we used only one cell line and at least three independent sgRNAs per gene. All sgRNAs of interest were validated in independent experiments in two cell models. For the analysis of phosphoproteomic changes after ATR inhibition, we used three independently grown biological replicates of the same rhabdomyosarcoma cell line. For in vivo testing, sample size was decided based on previous experience with the models. Animals euthanized before the end of the experiment, due to excessive tumor growth or loss of body weight, were included in the analysis.

### Reagents

All reagents were obtained from Carl Roth (Karlsruhe, Germany) unless otherwise indicated. Oligonucleotide primers were obtained from Eurofins Genomics (Luxemburg, Luxemburg, complete sequence in Supplementary Table 1). A list of antibodies and their catalog numbers can be found in Supplementary Table 2. AZD6738 (ceralasertib) was provided by Astra Zeneca (Cambridge, UK). BAY 1895344 (elimusertib) was provided by Bayer AG. All drugs were dissolved in Dimethylsulfoxid (DMSO) and stored at 10 mM concentrations at −20 °C.

### Plasmid constructs

Human *PAX3-FOXO1* cDNA was PCR-amplified and isolated from a plasmid gifted by Prof. Beat Schäfer. *PAX3-FOXO1* cDNA was cloned into pENTR1A (Thermo Fisher) using the restriction enzymes SalI and NotI (New England Biolabs) and cloned into a pInducer20 (Addgene #44012) using the Gateway strategy and the manufacturer’s protocol (Thermo Fisher). pLKO.1 shRNA plasmids targeting BRCA1 (TRCN0000009823, TRCN0000010305, TRCN0000039834), ATR (TRCN0000010301, TRCN0000039614, TRCN0000039615, TRCN0000039616) and control targeting GFP (shGFP) were obtained from the RNAi Consortium (Broad Institute). Plasmid containing an inducible shRNA targeting PAX3-FOXO1 (cloned in the pRSI backbone) were a kind gift from Prof. Beat Schäfer.

### Cell culture

Rh41, Kym1, and Rh18 cells were a kind gift from Prof. Simone Fulda. Rh5, RMS and KFR were a kind gift from Prof. Beat Schäfer. The remaining human tumor cell lines were obtained from the American Type Culture Collection (ATCC, Manassas, Virginia). The absence of *Mycoplasma sp*. contamination was determined using a Lonza (Basel, Switzerland) MycoAlert system. Rh4, Rh5, Rh30, Rh41, RMS, KFR, RD, T174, TE381.T, C2C12, 5838, A4573, CHP, JR, SB, SK-N-MC, TC-71 and HEK293T cell lines were cultured in Dulbecco’s Modified Eagle’s Medium (DMEM, Thermo Fisher, Waltham, Massachusetts, USA) supplemented with 10% fetal calf serum (Thermo Fisher) and penicillin/streptomycin (Thermo Fisher). CADO-ES1, Rh18, and Kym1 cells were cultured in Roswell Park Memorial Institute (RPMI)−1640 (Thermo Fisher) supplemented with 10% fetal calf serum and penicillin/streptomycin. Twice per week, cells were washed with phosphate-buffered saline (PBS), incubated in trypsin (Thermo Fisher) for five minutes sedimented at 500 g for 5 min and a fraction was cultured in fresh media. Cells were kept in culture for a maximum of 30 passages. Resuspended cells were counted by mixing 1:1 with 0.02 % trypan blue in a BioRad (Hercules, CA, USA) TC20 cell counter.

Human primary myoblasts were established from muscle biopsies obtained from M. vastus lateralis and M. triceps brachii. Volunteers were a 41F (Myo1), 32M (Myo2), 44F (Myo3), 52F (Myo4), and 21F (Myo5) who came to the hospital with a diagnosis (myalgia, cramps, myalgia, mialgya and family history of myopathy, respectively), but had no myopathology. All donors provided informed consent, and the myoblast isolation was done at the HELIOS Hospital Berlin Buch (Berlin, Germany) with the approval by the regulatory agencies (Ethics committee of Charité Universitätsmedizin Berlin, in compliance with the Declaration of Helsinki, approval number EA2/175/17). Myoblasts were grown in Skeletal Muscle Growth Medium (Provitro, Berlin, Germany) without antibiotics. Contamination of myoblast cultures with fibroblast was assessed by anti-desmin staining and was always below 5%.

### Lentiviral transduction

Lentivirus were produced as previously described^70^. In short, HEK293T cells were transfected using TransIT-LT1 (Mirus, Madison, Wisconsin, USA) in a 2:1:1 ratio of lentiviral plasmid, psPAX2, and pMD2.G plasmids following the TransIT-LT1 manufacturer’s protocol. Viral supernatant was collected 48 and 72 h after transfection, pooled, filtered, and stored at −80 °C. Cells were transduced for one day in the presence of 8 µg/mL polybrene (Sigma Aldrich).

### CRISPRa screening and sequencing

The genome-wide CRISPRa screen was performed as described in Konermann et al.^50^. Briefly, Rh4 cells were transduced with the lentiMPH v2 plasmid (Addgene #89308) and selected with hygromycin for 10 days (Thermo Fisher). Next, cells were transduced with the sgRNA library at a multiplicity of infection (MOI) of <0.3, ensuring at least 500 cells to be transduced with each sgRNA-encoding plasmid on average. After selection with blasticidin (Thermo Fisher) for 7 days, cells were separated in two groups, one group was incubated in the presence of AZD6738 at 750 nM concentration and the other group was incubated in the presence of DMSO. Genomic DNA was extracted and the sgRNA amplified using PCR and barcoded for Illumina sequencing. Sequencing was performed on a NextSeq500 with Mid Output, with a read length of 1 × 81 bp +8 bp Index and 20% PhiX Control v3. Samples were demultiplexed using flexbar^71^ and analyzed using MAGeCK (v. 0.5.6)^72^. Pathway analysis was performed using the R package msigdbr (R version 4.0.3; RStudio v1.3.1093; msigdbr v.7.4.1), providing a ranked list of genes and log-fold change and selecting the hallmark pathways from MSigDB^73,74^.

### Cell viability

Cell viability was assessed using CellTiter-Glo (Promega, Madison, Wisconsin, USA). Briefly, 1000 cells were seeded in white, flat-bottom, 96-well plates (Corning, Corning, NY, USA). After 24 h, drugs were added to the medium, and cells were incubated for 72 h. CellTiter-Glo luminescent reagent was added according to the manufacturers protocol, and the luminescence signal measured on a Synergy LX (Agilent, California, USA) with BioTek Gen5 (v3.08). To evaluate if a combination of drugs is synergistic, cells were simultaneously treated with varying concentrations of drugs, and cell viability was measured with CellTiter-Glo. Synergism scores were obtained using the R package SynergyFinder (v2.2.4)^75^.

### Immunoblotting

Whole-cell protein lysates were prepared by lysing cells in Radioimmunoprecipitation assay buffer (RIPA) supplemented with cOmplete Protease inhibitor (Roche, Basel, Switzerland) and PhosphStop (Roche). Protein concentrations were determined by bicinchoninic acid assay (BCA, Thermo Fisher). 10 µg of protein were denatured in Laemmli buffer at 95 °C for 5 min. Lysates were loaded onto 16% or 10% Tris-Glycin (Thermo Fisher) for gel electrophoresis depending on the protein sizes of interest. Proteins were transferred onto Polyvinylidenfluorid (PVDF) membranes (Roche), blocked with 5% dry milk for 1 h, and incubated with primary antibodies overnight at 4 °C, then secondary antibodies for 1 h at room temperature. Chemiluminescent signal was detected using Enhanced chemiluminescence (ECL) Western Blotting Substrate (Thermo Fisher) and a Fusion FX7 imaging system (Vilber Lourmat, Marne-la-Vallée, France) using ImageLab (v6.0.1). Quantification was performed with ImageJ (v.1.52a).

### Immunofluorescence

Cells were grown at the desired confluency on a glass coverslide for 24 h (micronuclei quantification) and treated with 1000 ng/mL doxycycline for another 48 h (for the corresponding experiment). Cells were washed with PBS three times and fixed for 10 min with 4% paraformaldehyde, washed with PBS three times and permeabilized with PBS containing 0.1% Triton-X100. For micronuclei detection, cells were mounted on a slide with DAPI-containing mounting media (Vectashield, Vec-H-1000). For immunofluorescence, cells were blocked for 40 min with 5% BSA in PBS, incubated overnight at 4 °C with the primary antibody, washed three times with PBS-T (0.05% Tween-20 in PBS), incubated for 1 h in the dark at room temperature with the secondary antibody, washed three times with PBS-T and mounted on a slide with DAPI-containing mounting media. Cells were imaged using an ECHO Revolve microscope and quantified using ImageJ (v.1.52a).

### RT-qPCR

RNA from cell lines was extracted using RNeasy mini kit (QIAGEN). Synthesis of cDNA was performed using Transcription First Strand cDNA Synthesis kit (Roche). 50 ng of cDNA were combined with the corresponding primers (Supplementary Table 1), and SG qPCR Master Mix (Roboklon, Berlin, Germany), keeping the mixture and cycling conditions recommended by the manufacturer. DNA content was measured using a CFX Connect Real-Time PCR detection system (BioRad) with the software CFX Manager (v3.1).

### Fluorescence-activated cell sorting (FACS)

For cell cycle analysis, cells were incubated with 5-Ethynyl-2´-deoxyuridine (EdU) for 2 h and fluorescent labeling was performed with the Click-IT EdU Alexa Fluor 488 Flow Cytometry Assay kit (Thermo Fisher), according to the manufacturer’s description. Terminal deoxynucleotidyl transferase dUTP nick end labeling (TUNEL) was performed using the APO-BrdU TUNEL Assay Kit (Thermo Fisher), according to the manufacturer’s descriptions. Cell death was assessed by measuring caspase 3 cleavage using a CellEvent Caspase3/7 Green Flow Cytometry kit (Thermo Fisher), according to the manufacturer’s descriptions. Stained cells were measured on a BD LSR Fortessa flow cytometer (BD Biosciences, Franklin Lakes, NJ, USA) with the BD FACS Diva (v8.0.1) and analyzed with FlowJo (v10.6.2).

### Homologous recombination activity assay

All the plasmids were obtained from Addgene (pDRGFP #26475pCBA-SceI #26477; pCAG-FALSE #89689; pCAGGS-mCherry #41583). The protocol was adapted from the plasmid depositors’^76,77^. Briefly, cells were co-transfected with pCBA-SceI and pDRGFP to analyze homologous recombination. As a negative control, pCBA-SceI was substituted with the empty backbone pCAG-FALSE. Transfection efficiency was calculated using cells transfected with pCAGGS-mCherry. Two days after transfection, cells were trypsinized, washed twice with PBS and fluorescence measured with flow cytometry. When necessary, cells were treated with 750 nM AZD6738 for five days prior to flow cytometry analysis.

### Phosphoproteomics sample preparation

Rh30 cells were cultured for two weeks in the presence of stable isotope labeling with amino acids (SILAC) media in DMEM, 10% dialyzed fetal calf serum, 1% Proline, 1% Glutamine, 0.025% ^8^Lysine, and ^10^Arginine (“Heavy”) or ^0^Lysine and ^0^Arginine (“Light”). After labeling, cells were incubated in the presence of AZD6738 750 nM or DMSO for two hours in biological triplicates. Cells were harvested, resuspended and combined in 400 µL of 8 M urea and 0.1 M Tris-HCl, pH 8. Proteins were reduced in 10 mM dithiothreitol (DTT) at room temperature for 30 min and alkylated with 50 mM iodoacetamide (IAA) at room temperature for 30 min in the dark. Proteins were first digested by lysyl endopeptidase (LysC) (Wako Pure Chemical Industries, Ltd., Osaka, Japan) at a protein-to-LysC ratio of 100:1 (w/w) at room temperature for 3 h. Then, the sample solution was diluted to final concentration of 2 M urea with 50 mM ammonium bicarbonate (ABC). Trypsin (Promega) digestion was performed at a protein-to-trypsin ratio of 100:1 (w/w) under constant agitation at room temperature for 16 h. Tryptic digests corresponding to 200 µg protein per condition were desalted with big C18 Stage Tips packed with 10 mg of ReproSil‐Pur 120 C18‐AQ 5 µm resin (Dr. Maisch GmbH, Ammerbuch, Germany). Peptides were eluted with 200 µL of loading buffer (80% ACN (v/v) and 6% TFA (v/v). Phosphopeptides were enriched using a microcolumn tip packed with 0.5 mg of TiO_2_ (Titansphere, GL Sciences, Tokyo, Japan)^78^. The TiO_2_ tips were equilibrated with 20 μL of the loading buffer via centrifugation at 100 × *g*. 50 μL of the sample solution was loaded on a TiO_2_ tip via centrifugation at 100 × *g* and this step was repeated until the sample solution was loaded. The TiO_2_ column was washed with 20 μL of the loading buffer, followed by 20 μL of washing buffer (50% ACN (v/v) and 0.1% TFA (v/v)). The bound phosphopeptides were eluted using successive elution with 30 μL of elution buffer 1 (5% ammonia solution), followed by 30 μL of elution buffer 2 (5% piperidine)^79^. Each fraction was collected into a fresh tube containing 30 μL of 20% formic acid. 3 μL of 100% formic acid was added to further acidify the samples. The phosphopeptides were desalted with C18 Stage Tips prior to nanoLC-MS/MS analysis.

### NanoLC-MS/MS analysis

Peptides were separated on a 2 m monolithic column (MonoCap C18 High Resolution 2000 (GL Sciences), 100 µm internal diameter × 2000 mm at a flow rate of 300 nL/min with a 5–95% acetonitrile gradient on an EASY-nLC II system (Thermo Fisher Scientific). 240 min gradient was performed for phosphoproteome analyses. A Q Exactive plus instrument (Thermo Fisher Scientific) was operated in the data dependent mode with a full scan in the Orbitrap followed by top 10 MS/MS scans using higher-energy collision dissociation (HCD). For whole proteome analyses, the full scans were performed with a resolution of 70,000, a target value of 3 × 10^6^ ions, and a maximum injection time of 20 ms. The MS/MS scans were performed with a 17,500 resolution, a 1 × 10^6^ target value, and a 20 ms maximum injection time. For phosphoproteome analyses, the full scans were performed with a resolution of 70,000, a target value of 3 × 10^6^ ions, and a maximum injection time of 120 ms. The MS/MS scans were performed with a 35,000 resolution, a 5 × 10^5^ target value, and a 160 ms maximum injection time. Isolation window was set to 2 and normalized collision energy was 26.

Raw data were analyzed and processed using MaxQuant (v1.5.1.2)^80^. Search parameters included two missed cleavage sites, fixed cysteine carbamidomethyl modification, and variable modifications including L-[^13^C_6_,^15^N_4_]-arginine, L-[^13^C_6_,^15^N_2_]-lysine, methionine oxidation, N-terminal protein acetylation, and asparagine/glutamine deamidation. In addition, phosphorylation of serine, threonine, and tyrosine were searched as variable modifications for phosphoproteome analysis. The peptide mass tolerance was 6 ppm for MS scans and 20 ppm for MS/MS scans. Database search was performed using Andromeda^81^ against uniprot-human 2014-10 with common contaminants. False discovery rate (FDR) was set to 1% at both peptide spectrum match (PSM) and protein level. The ‘re-quantify’ and ‘match between runs’ functions were enabled. Phosphorylation sites were ranked according to their phosphorylation localization probabilities (P) as class I (*P* > 0.75), class II (0.75 > *P* > 0.5), and class III sites (*P* < 0.5), and only class I sites were used for further analyses. Data normalization was performed using the default settings of the R package DEP^82^. In short, peptides not identified in at least two replicates in both conditions were removed. Intensity values were normalized based on the variance stabilizing transformation, and missing values were imputed using random draws from a Gaussian distribution centered around a minimal value (*q* = 0.01). For pathway enrichment analysis, we used a single sample gene set enrichment analysis (ssGSEA) as previously described^38^, ranking genes according to their fold change. For gene ontology (GO) analysis, we followed the ClusterProfiler R package (v3.16.1)^83^. *P*-values were calculated using hypergeometric distribution (one-sided Fisher exact test) and corrected for multiple comparisons (Holm–Bonferroni method), selecting phosphopeptides with a fold change >1 or <−1 and a FDR < 0.01, and reporting the top 10 GO terms enriched in the subset.

### Patient-derived xenograft (PDX) treatment

The establishment of PDX models was conducted as previously described^84^ in collaboration with Experimental Pharmacology & Oncology GmbH (EPO, Berlin, Germany). Briefly, a tumor fragment was serially transplanted in mice at least three times prior to the experiments. All experiments were conducted according to the institutional animal protocols and the national laws and regulations and approved by the Charité University Medicine and MSKCC IACUC. Fusion status was determined by PCR at time of diagnosis. Tumor fragments from rhabdomyosarcoma patients were transplanted into NOD.Cg-*Prkdc*^*scid*^ *Il2rg*^*tm1Sug*^/*JicTac* female mice between 6 and 8 weeks old (Taconic, Rensselaer, NY, USA) or NSG-H (NOD.Cg-*Prkdc*^*scid*^ *Hprt*^*em1Mvw*^ *Il2rg*^*tm1Wjl*^/*MvwJ*; for the PAX7-FOXO1 ARMS PDXs) male and female mice mix between 6 and 8 weeks old. Animals were IVC housed under sterile and standardized conditions (22 °C +/−1 °C, 50% relative humidity, 12-hour light-dark cycle, autoclaved food, bedding material and tap water ad libitum). Tumor growth was monitored with caliper measurements. Tumor volume was calculated with the formula length x width^2^/2. PDX were serially transplanted in mice at least three times prior to the experiments. Mice were randomized into four groups with at least 3 mice to receive AZD6738 (50 mg/kg day, oral), olaparib (50 mg/kg day, oral), a combination of AZD6738 and olaparib, or vehicle. For in vivo treatment, AZD6738 was dissolved in DMSO at 62.5 mg/ml and mixed 1:10 in 40% propylene glycol and 50% sterile water, resulting in a final AZD6738 concentration of 6.25 mg/ml. Olaparib was dissolved in 4% DMSO, 30% polyethylene glycol 300 and sterile water. For the BAY 1895344 study, mice were administered 40 mg/kg body weight on a 3 days on/ 4 days off regime twice daily (orally). BAY 1895344 was dissolved in 60% polyethylene glycol 400, 10% ethanol, and 30% water to a 4 mg/ml solution. Ifosfamide was dissolved in 0.9% sodium chloride and administered intravenously at a 50 mg/mL concentration up to 80 mg/kg body weight per day twice weekly. Vincristine was dissolved in 0.9% sodium chloride and administered daily intravenously at 1 mg/mL up to 1 mg/kg body weight per day. Solutions in which the drugs were dissolved were used as vehicle controls respectively. Mice were sacrificed by cervical dislocation once the tumor volume exceeded 2000 mm^3^ or body weight loss was higher than 10%. For the toxicity study, blood was drawn, and blood count was analyzed by Synlab (Berlin, Germany). Organ tissue was collected, fixed with formalin and embedded into paraffin, sliced, and stained with hematoxylin & eosin following the standard diagnostics protocol. For immunohistochemistry staining of cleaved caspase 3 and Ki67, snap frozen tumor fragments were cut and stained following the standard protocol using the antibodies listed in Supplementary Table 2.

### RNA-seq of ATR inhibitor resistant cells

To generate cells resistant to ATR inhibitors, cells were cultured with an IC10 of the corresponding ATR inhibitor for at least three passages. The concentration was doubled for a total of four months. At that point, pellets were collected and prepared for RNA-seq using TruSeq Standard mRNA library prep according to the manufacturer’s instructions. Samples were sequenced using a NextSeq500 mid output using pair ended reads (2x75bp). Reads were filtered by sequence quality using trimGalore!, aligned to the reference genome (hg19 [https://www.ncbi.nlm.nih.gov/assembly/GCF_000001405.13/]) using STAR^85^ and counted using HTSeq^86^. For pathway analysis, we used the package gage and selected the hallmark pathways from MSigDB^73,74^.

### Statistics and reproducibility

All statistical tests were done using GraphPrism7 (student’s two-sided t-test) or were part of the R package used for the analysis (MAGeCK-VISPR, DEP, CePa, msigdbr, gage). All computational analyses were performed using Python 3.7 (MAGeCK-VISPR v.0.5.6, TrimGalore! v.0.6.1, STAR v.2.7.9a, HTSeq v.1.99.2) or RStudio v.1.3.1093 (R v.4.0.3, biomaRt v.2.44.4, CePa v.0.7.0, clusterProfiler v.3.16.1, DEP v.1.10.0, gage v.2.38.3, msigdbr v.7.4.1, org.Hs.eg.db v.3.11.4, synergyfinder v.2.2.4, tidyverse v.1.3.1).

Western immunoblots (in Figs. 2a, b, h, i, 3d, e, 4a, h, 5g, h, l, 6g, h, Supplementary Figs. 2a, e, 4a, 5a, 6a, 8f, g, and 10a) were done in one independent experiment, but include different biologically independent cell models (Figs. 2h, i, 3d, e, 5g, h and Supplementary Figs. 6f, g, and Fig. 5a), two independent small molecule inhibitors (Figs. 2a, b, h, i, 3d, e, and 6g, h) or include at least three independent shRNA or sgRNA, respectively (Figs. 4h, 5g, h, l and Supplementary Figs. 2a, e, 4a, 8f, g). Immunofluorescence experiments (Figs. 2d, 4b) were repeated three times. For micronucleation (Fig. 2d, e), each replicate includes 50 cells. Histochemistry experiments (Fig. 7h and Supplementary Fig. 10a, p) were performed once. Quantification of immunohistochemistry (Fig. 7i, j) was performed in 10 representative 275 µm × 275 µm sections per group.

### Reporting summary

Further information on research design is available in the Nature Research Reporting Summary linked to this article.

## Supplementary information

Supplementary Information

Reporting Summary

## Data Availability

The reference genome hg19 [https://www.ncbi.nlm.nih.gov/assembly/GCF_000001405.13/] used in this study is publicly available. The proteomics data which support the findings in this study have been deposited in the ProteomeXchange Consortium via jPOST partner repository with the dataset identifier JPST001683 and the accession code identifier PXD035131. The CRISPR reads generated in this study are available from the NCBI Sequence Read Archive (SRA) Sequence Read Archive (SRA) under the BioProject code PRJNA856804. The RNA-seq reads generated in this study have been deposited in the SRA, accessible under the BioProject code PRJNA856799. Source data are provided with this paper.

## Source data

Source Data
