# Supplementary material for: Therapeutic targeting of ATR in alveolar rhabdomyosarcoma
Source: Nat Commun. 2022 Jul 25;13:4297. doi: 10.1038/s41467-022-32023-7 (PMC9314382; doi:10.1038/s41467-022-32023-7)
Supplement: Supplementary file 1 — Supplementary Information [file 41467_2022_32023_MOESM1_ESM.pdf]

Supplementary Information for

Therapeutic targeting of ATR in alveolar rhabdomyosarcoma

Heathcliff Dorado García, Fabian Pusch, Yi Bei, Jennifer von Stebut, Glorymar Ibáñez, Kristina Guillan, Koshi Imami, Dennis Gürgen, Jana Rolff, Konstantin Helmsauer, Stephanie Meyer-Liesener, Natalie Timme, Victor Bardinet, Rocío Chamorro González, Ian C. MacArthur, Celine Y. Chen, Joachim Schulz, Antje M. Wengner, Christian Furth, Birgit Lala, Angelika Eggert, Georg Seifert, Patrick Hundsoerfer, Marieluise Kirchner, Philipp Mertins, Matthias Selbach, Andrej Lissat, Frank Dubois, David Horst, Johannes H. Schulte, Simone Spuler, Daoqi You, Filemon Dela Cruz, Andrew L. Kung, Kerstin Haase, Michela di Virgilio, Monika Scheer, Michael V. Ortiz, Anton G. Henssen*

*Corresponding author. Email: henssenlab@gmail.com

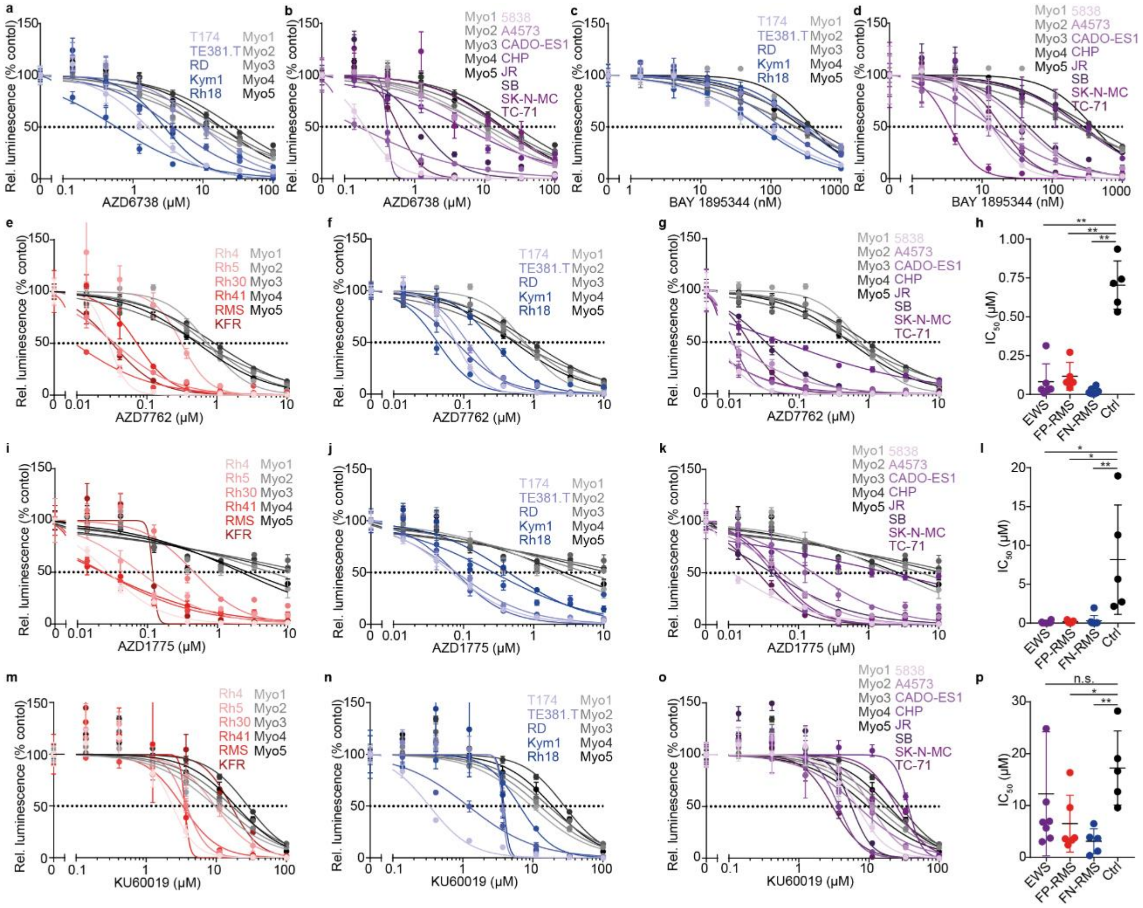

Supplementary Fig. 1. Effects of DNA Damage Response inhibitors in RMS and EWS cell lines (a-b) Dose-response curves of cell viability for FN-RMS (a) and EWS cell lines (b) treated with the ATR inhibitor AZD6738 compared to primary myoblasts (*n*=3). **(c-d)** Dose-response curves of cell viability for FN-RMS (c) and EWS cell lines (d) treated with the ATR inhibitor BAY 1895344 compared to primary myoblasts (*n*=3). **(e-g)** Dose-response curves of cell viability for FP-RMS (e) FN-RMS (f) and EWS cell lines (g) treated with the CHK1/2 inhibitor AZD7762 compared to primary myoblasts (*n*=3). **(h)** IC_{50} values for FP-RMS, EWS, FN-RMS and Ctrl cells treated with AZD7762 (P=6.95x10^{-8}; 3.29x10^{-5}; 8.45x10^{-5} for EWS, FP-RMS and FN-RMS vs Ctrl, respectively; from left to right, n=8, 6, 5 and 5 biologically independent cells). **(i-k)** Dose-response curves of cell viability for FP-RMS (i) FN-RMS (j) and EWS cell lines (k) treated with the WEE1 inhibitor AZD1775 compared to primary myoblasts (*n*=3). **(l)** IC_{50} values for FP-RMS, EWS, FN-RMS and Ctrl cells treated with AZD1775 (P=0.008; 0.020; 0.035 for EWS, FP-RMS and FN-RMS vs Ctrl, respectively; from left to right, n=8, 6, 5 and 5 biologically independent cells). **(m-o)** Dose-response curves of cell viability for FP-RMS (m) FN-RMS (n) and EWS cell lines (o) treated with the ATM inhibitor KU60019 compared to primary myoblasts (*n*=3). **(p)** IC_{50} values for FP-RMS, EWS, FN-RMS and Ctrl cells treated with KU60019 (P=0.421; 0.020; 0.030 for EWS, FP-RMS and FN-RMS vs Ctrl, respectively; from left to right, n=8, 6, 5 and 5 biologically independent cells). All statistical analyses correspond to two-sided student's t-test; data presented as mean value ± error bars representing standard deviation. Source data are provided as a Source Data file.

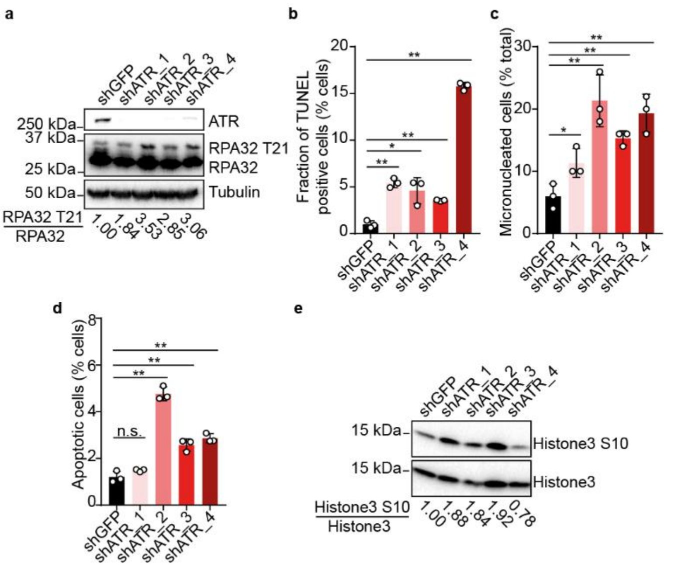

Supplementary Fig. 2. ATR knockdown leads to increased DNA damage and genomic instability. **(a)** Western immunoblot of ATR and RPA32 phosphorylation at T21 in Rh4 cells expressing shRNAs targeting ATR compared to shGFP expressing cells. **(b)** Quantification of TUNEL signal in Rh4 cells expressing shRNAs targeting ATR compared to shGFP expressing cells. (n = 3; from left to right, P = 2.74×10^{-4}; 0.012; 3.93×10^{-4}; 1.52×10^{-6}). **(c)** Fraction of micronucleated Rh4 cells expressing shRNAs targeting ATR compared to shGFP expressing cells. (n = 3, with 50 nuclei counted per replicate; P = 0.039; 0.005; 0.002; 0.003). **(d)** Fraction of apoptotic Rh4 cells expressing shRNAs targeting ATR compared to shGFP expressing cells. (n = 3; from left to right, P = 0.121; 8.61×10^{-5}; 0.003; 7.72×10^{-4}). **(e)** Western immunoblot of Histone 3 phosphorylation at S10 in Rh4 cells expressing shRNAs targeting ATR compared to shGFP expressing cells. All statistical analyses correspond to two-sided student's t-test; data presented as mean value ± error bars representing standard deviation. Source data are provided as a Source Data file.

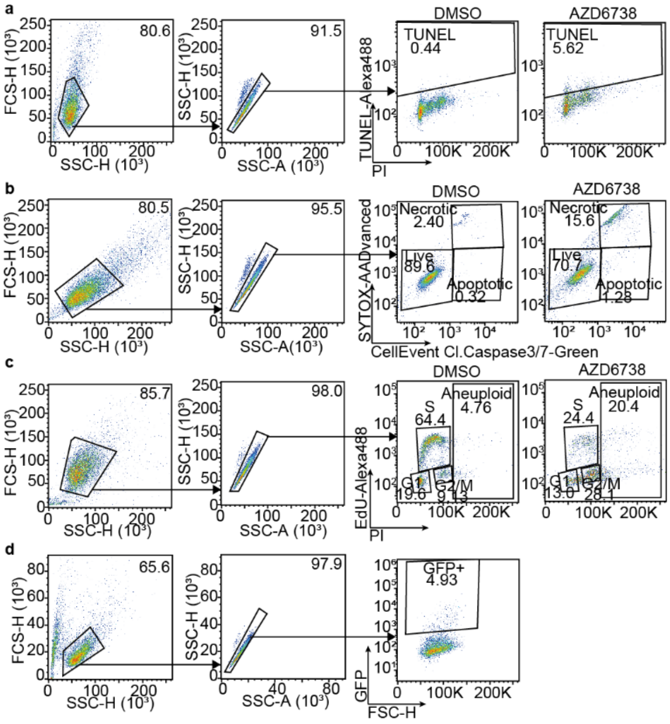

Supplementary Fig. 3. Representative FACS gating used in the study. (**a)** Representative gating of unrepaired DSBs measured by TUNEL. (**b)** Representative gating of cells stained for Caspase3/7 cleavage and SYTOX. **(c)** Representative gating of cell cycle phase distribution and aneuploidy as measured after EdU and propidium iodide co-staining. **(d)** Representative FACS gating of cells with active HR activity, measured as GFP reconstitution based on repair of an SceI-mediated DNA lesion via homologous recombination. FSC-H: Forward scatter (height), SSC-H: Side scatter (height), SSC-A: Side scatter (area).

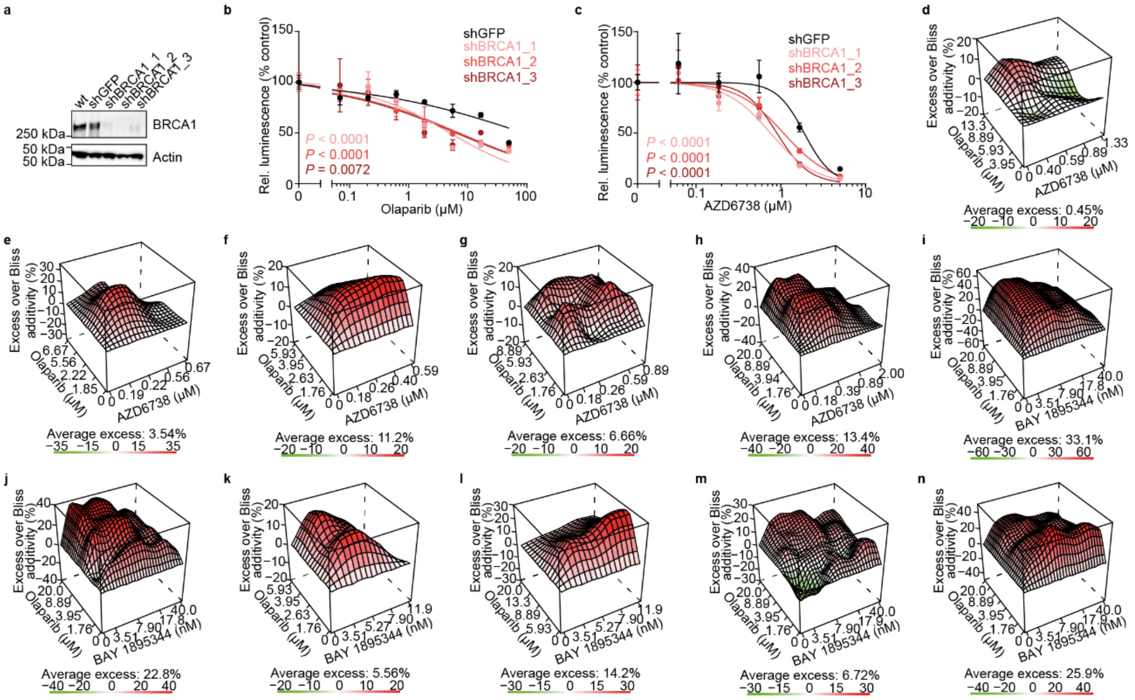

Supplementary Fig. 4. ATR inhibition synergizes with olaparib in fusion-positive rhabdomyosarcoma cell lines. (a) Western immunoblot of BRCA1 in Rh4 cells of stably expressing shRNAs targeting BRCA1 (shRNA targeting GFP was used as a control). **(b)** Dose-response curves for Rh4 stably expressing different shRNAs targeting BRCA1 and treated with PARP1 inhibitor olaparib (shRNA targeting GFP was used as a control) (n = 3). **(c)** Dose-response curves for Rh4 transduced with different shRNA targeting BRCA1 treated with ATR inhibitor AZD6738 (shRNA targeting GFP was used as a control) (n = 3). **(d-h)** Excess over Bliss analysis of combined treatment with olaparib and AZD6738 in Rh5 (d), Rh30 (e), Rh41 (f), RMS (g) and KFR (h) cells (*n* = 3). **(i-n)** Excess over Bliss analysis of combined treatment with olaparib and BAY 1895344 in Rh4 (i) Rh5 (j), Rh30 (k), Rh41 (l), RMS (m) and KFR (n) cells (*n* = 3). All statistical analyses correspond to two-sided student's t-test; data presented as mean value ± error bars representing standard deviation. Source data are provided as a Source Data file.

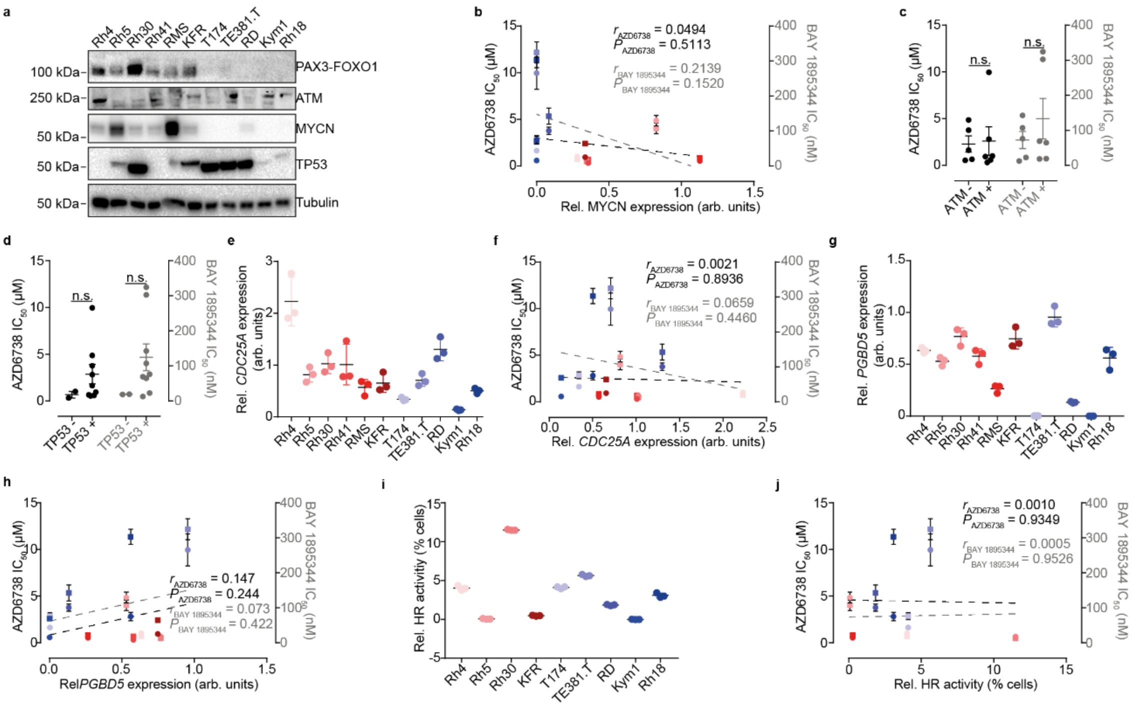

Supplementary Fig. 5. Molecular factors associated with ATR inhibitor sensitivity in rhabdomyosarcoma cells. (**a**) Western Immunoblot of PAX3-FOXO1, MYCN, ATM and TP53 in rhabdomyosarcoma cell lines. (**b**) Correlation of MYCN protein levels and IC$_{50}$ values for AZD6738 and BAY 1895344 (n=3 biologically independent measurements of IC$_{50}$ values). (**c-d**) Comparison of IC$_{50}$ values for AZD6738 and BAY 1895344 for ATM (c*;* n=5 and n=6 for ATM- and ATM+, respectively; P = 0.869; 0.392) and TP53 (d; n=2 and n=9 for TP53- and TP53+, respectively; P = 0.356; 0.240). **(e)** mRNA expression levels of *CDC25A* in rhabdomyosarcoma cell lines (n = 3). **(f)** Correlation of *CDC25A* expression and IC$_{50}$ values for AZD6738 and BAY 1895344 (n=3 biologically independent measurements of IC$_{50}$ values)**. (g)** mRNA expression levels of *PGBD5* in rhabdomyosarcoma cell lines (n = 3). **(h)** Correlation of *PGBD5* expression and IC$_{50}$ values for AZD6738 and BAY 1895344 (n=3 biologically independent measurements of IC$_{50}$ values). **(i)** Quantification of HR activity in RMS cells (n = 3). **(j)** Correlation of HR activity and IC$_{50}$ values for AZD6738 and BAY 1895344 (n=3 biologically independent measurements of IC$_{50}$ values). All statistical analyses correspond to two-sided student's t-test; data presented as mean value ± error bars representing standard deviation. Source data are provided as a Source Data file.

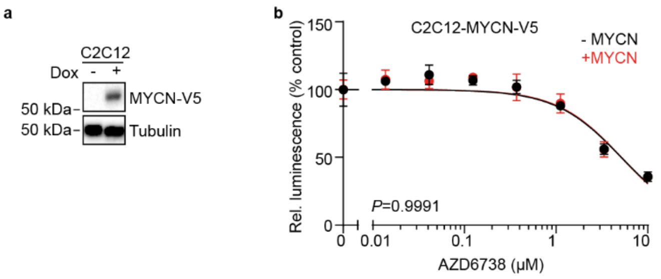

Supplementary Fig. 6. Ectopic expression of MYCN has no effect in ATR inhibitor sensitivity in C2C12 myoblasts. (a) Western immunoblotting of MYCN in C2C12 after induction with doxycycline (1000 ng/ml for 48h). **(b)** Dose-response curves in C2C12 cells treated with ATR inhibitor AZD6738 after doxycycline-induced ectopic expression of MYCN (*n*=3; error bars represent standard error of the mean). All statistical analyses correspond to two-sided student's t-test; data presented as mean value ± error bars representing standard deviation. Source data are provided as a Source Data file.

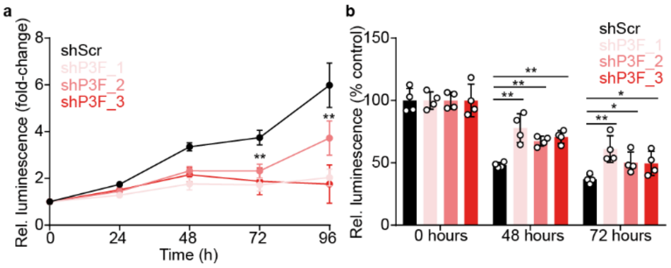

Supplementary Fig. 7. PAX3-FOXO1 knockdown reduces sensitivity to ATR inhibition (a) Proliferation of Rh4 cells over time after doxycycline-induced knockdown of PAX3-FOXO1 (n=8; *P* = 1.49x10^{-9}; 2.11x10^{-7}; 1.20x10^{-6}; 2.76x10^{-8}; 1.06x10^{-4}; 1.67x10^{-7}). **(b)** Cell viability reduction of Rh4 cells treated with AZD6738 (750 nM) after doxycycline-induced knockdown of PAX3-FOXO1 (*n*=4; *P* = 0.002; 0.001; 1.88x10^{-4}; 0.005; 0.027; 0.048). All statistical analyses correspond to two-sided student's t-test; data presented as mean value ± error bars representing standard deviation. Source data are provided as a Source Data file.

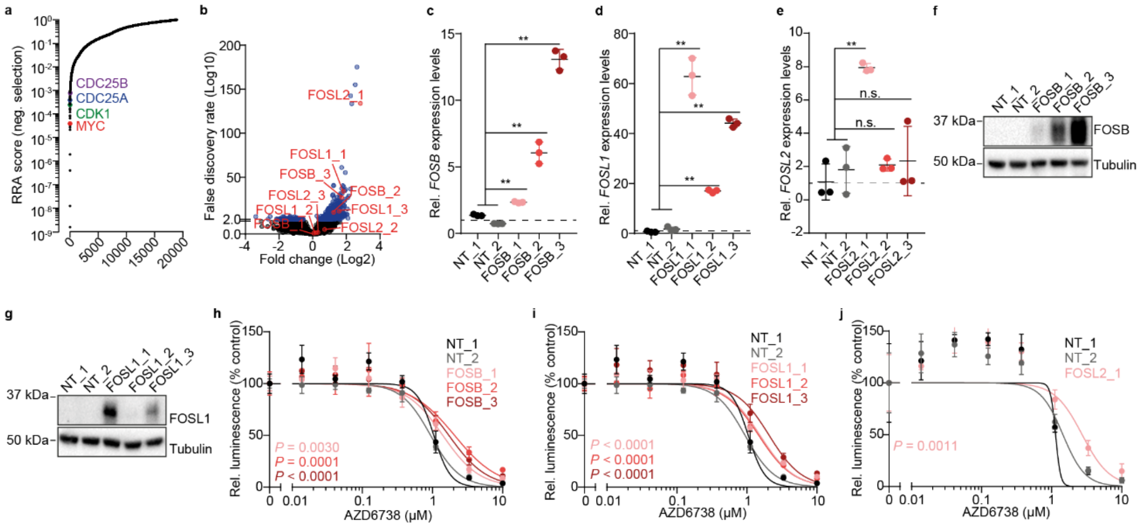

Supplementary Fig. 8. *FOSB*, *FOSL1* and *FOSL2* expression reduces sensitivity to ATR inhibition in rhabdomyosarcoma cells. (a) Waterfall plot showing the negative robust rank aggregation (RRA) score of sgRNAs in Rh4 cells incubated in the presence of AZD6738 for 9 days compared to DMSO treated cells as analyzed using MAGeCK. **(b)** Volcano plot showing the changes in sgRNA enrichment in Rh4 cells incubated in the presence of AZD6738 for 9 days compared to DMSO treated cells as analyzed using MAGeCK. In red, all the sgRNA corresponding to *FOSB*, *FOSL1* and *FOSL2*. **(c-e)** *FOSB* (c; n=3 independent experiments; P = 4.7x10^{-4}, P = 2.9x10^{-6} and P = 5.1x10^{-9}), *FOSL1* (d; n=3 independent experiments; P = 1.2x10^{-7}, P = 1.6x10^{-8} and P = 2.2x10^{-10}) and *FOSL2* (e; n=3 independent experiments; P = 4.2x10^{-5}, P = 0.400 and P = 0.430) mRNA expression measured using RT-qPCR in Rh4 cells expressing dCas9, lentiMPH and sgRNAs targeting *FOSB*, *FOSL1* or *FOSL2* (n = 3). **(f-g)** Western immunoblot of FOSB (f) and FOSL1 (g) in Rh4 cells stably expressing dCas9, lentiMPH and sgRNAs targeting *FOSB* and *FOSL1*, respectively. **(h-j)** Relative cell viability of Rh4 cells stably expressing dCas9, lentiMPH and sgRNAs targeting *FOSB* (h), *FOSL1* (i) and *FOSL2* (j) in the presence and absence of AZD6738 (n = 3). All statistical analyses correspond to two-sided student's t-test; data presented as mean value ± error bars representing standard deviation. Source data are provided as a Source Data file.

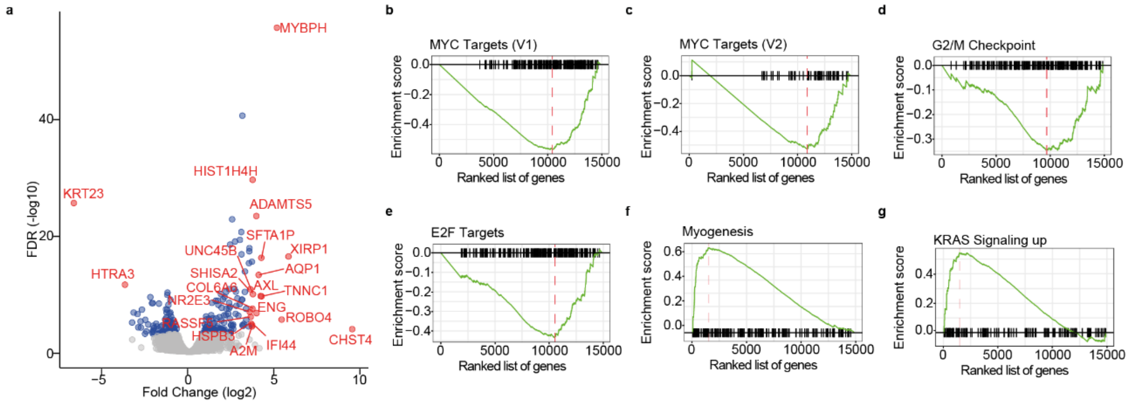

Supplementary Fig. 9. RAS-MAPK pathway is activated in Rh4 cells resistant to ATR inhibitors. (a) Volcano plot showing differentially expressed genes in cells incubated for 4 months with the ATR inhibitor AZD6738 vs a control population (red, top 20 differentially expressed genes). **(b-f)** GSEA plots showing enrichment of genes belonging to the MYC targets V1 (b), MYC targets V2 (c), G2/M checkpoint (d), E2F targets (e), myogenesis (f) and KRAS signaling up (g) pathways according to the GSEA hallmark pathways. Source data are provided as a Source Data file.

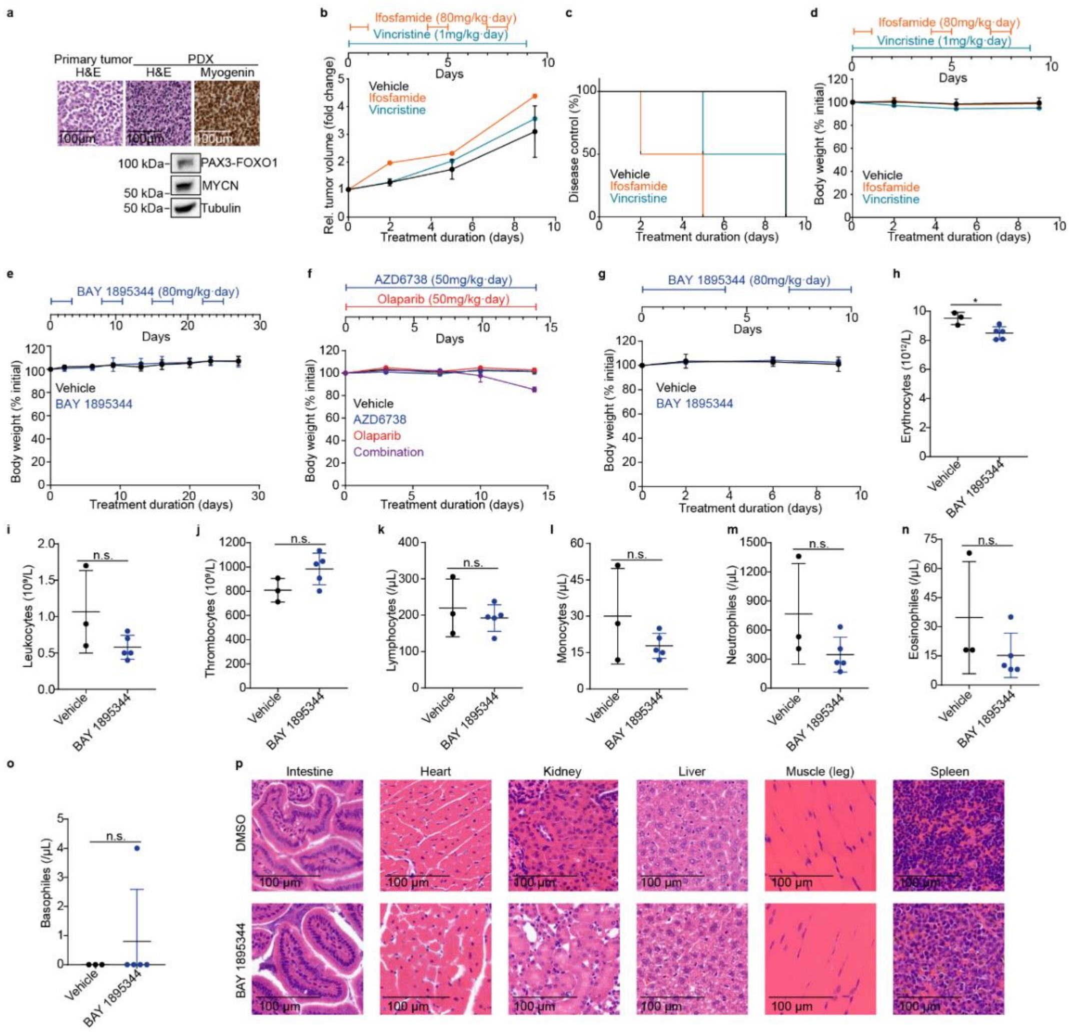

Supplementary Fig. 10. *In vivo* treatment with ATR inhibitors has no remarkable toxicity in mice harboring ARMS PDX models. (a) Representative histological images and western immunoblot for PAX3-FOXO1 and MYCN for the ARMS PDX model used. **(b)** Tumor volume change of the ARMS PDX and treated with ifosfamide or vincristine as compared to control (n=3 mice for vehicle, n=2 for treatments; top, timeline of the drug schedule). **(c)** Kaplan Meier curve showing tumor doubling time after treatment. **(d)** Body weight over time of mice harboring the ARMS PDX model and treated with ifosfamide, vincristine or control (n=3 mice for vehicle, n=2 for treatments). **(e)** Body weight over time of mice harboring the rhabdomyosarcoma PDX model and treated with BAY 1895344 or a vehicle control. (n=7). **(f)** Body weight over time of mice harboring the rhabdomyosarcoma PDX and treated with AZD6738, olaparib, both or a vehicle control (n=4). **(g)** Body weight over time of NOG mice treated with BAY 1895344 or a vehicle control. (n=3 for vehicle, n=5 for BAY 1895344 treated mice). **(h-o)** Blood counts for NOG mice treated with BAY 1895344 or vehicle, including erythrocytes (h; P=0.019), leukocytes (i; P=0.109), thrombocytes (j; P=0.093), lymphocytes (k; P=0.512), monocytes (l; P=0.217), neutrophiles (m; P=0.136), eosinophiles (n; P=0.212) and basophiles (o; P=0.482) (n=3 for vehicle, n=5 for BAY 1895344 treated mice for all figures from h to o). (p) Representative hematoxylin and eosin (H&E) histological images of mice organs after treatment with BAY 1895344 or vehicle. All statistical analyses correspond to two-sided student's t-test; data presented as mean value ± error bars representing standard deviation. Source data are provided as a Source Data file.

Primer	Sequence (5’-3’)
**CDC25A_Fwd**	ACC GTC ACT ATG GAC CAG C
**CDC25A_Rv**	TTC AGA GCT GGA CTA CAT CC
**FOSB_Fwd**	GTG AGA GAT TTG CCG GGC TC
**FOSB_Rv**	AGA GAG AAG CCG TCA GGT TG
**FOSL1_Fwd**	GCC CAC TGT TTC TCT TGA GC
**FOSL1_Rv**	GAT GGA GAG TGT GGC AGT GA
**FOSL2_Fwd**	GCC CAG TGT GCA AGA TTA GC
**FOSL2_Rv**	GGG CTC CTG TTT CAC CAC TA
**HPRT1_Fwd**	TGA CAC TGG CAA AAC AAT GCA
**HPRT1_Rv**	GGT CCT TTT CAC CAG CAA GCT
**PGBD5_Fwd**	CAG CCT CTG GGT CAG ACA AT
**PGBD5_Rv**	GCT TAT TCT TCA GCG CAT CC

Supplementary Table 1. Oligonucleotide sequences used in the manuscript.

Antibody	Company	Catalog number	Dilution
**Actin**	Cell Signaling Technology	3700	1:1000 in 5% milk in TBS
**ATM**	Cell Signaling Technology	92356	1:1000 in 5% milk in TBS
**ATR**	Cell Signaling Technology	13934	1:1000 in 5% milk in TBS
**BRCA1**	Merck Millipore	OP92-100UG	1:5000 in 5% milk in TBS
**BRCA1 S1524**	Bethyl Laboratories	A300-001A	1:1000 in 5% BSA in TBS
**ClCas3**	Cell Signaling Technology	9664	1:2000 in 3% BSA in PBS
**c-Raf**	Cell Signaling Technology	9422	1:1000 in 5% milk in TBS
**c-Raf S338**	Cell Signaling Technology	9427	1:1000 in 5% BSA in TBS
**ERK1/2**	Cell Signaling Technology	4695	1:1000 in 5% milk in TBS
**ERK1/2 T202/T204**	Cell Signaling Technology	4370	1:1000 in 5% BSA in TBS
**FOSB**	Cell Signaling Technology	2251	1:1000 in 5% milk in TBS
**FOSL1**	Cell Signaling Technology	5281	1:1000 in 5% milk in TBS
**FOXO1 (**And **PAX3-FOXO1)**	Santa Cruz Biotechnology	sc-374427	1:500 in 5% milk in TBS
**Histone 2A.X S139**	Merck Millipore	05-636	1:500 in 5% FBS in TBS-T
**Histone 3**	Cell Signaling Technology	4499	1:1000 in 5% milk in TBS
**Histone 3 S10**	Cell Signaling Technology	3377	1:1000 in 5% BSA in TBS
**Ki67**	Thermo Fisher Scientific	MA5-14520	1:20 in 3% BSA in PBS
**MYCN**	Santa Cruz Biotechnology	sc-53993	1:500 in 5% milk in TBS
**RPA32 T21**	Abcam	ab61065	1:10000 in 5% BSA in TBS
**TP53**	Santa Cruz Biotechnology	sc-98	1:500 in 5% milk in TBS
**Tubulin**	Cell Signaling Technology	3873	1:1000 in 5% milk in TBS
**Anti-mouse-HRP**	Thermo Fisher Scientific	62-6520	1:1000 in 5% milk or BSA in TBS
**Anti-rabbit-HRP**	Thermo Fisher Scientific	G-21234	1:1000 in 5% milk or BSA in TBS
**Anti-mouse-Alexa488**	Dianova	715-096-150	1:1000 in 5% FBS in TBS-T

Supplementary Table 2. Antibodies used in the manuscript, including dilution.
