# Supplementary material for: Therapeutic targeting of ATR in alveolar rhabdomyosarcoma
Source: Nat Commun. 2022 Jul 25;13:4297. doi: 10.1038/s41467-022-32023-7 (PMC9314382; doi:10.1038/s41467-022-32023-7)
Supplement: Supplementary file 2 — Reporting Summary [file 41467_2022_32023_MOESM2_ESM.pdf]

nature portfolio

Corresponding author(s): Anton G. Henssen

Last updated by author(s): Jun 27, 2022

Reporting Summary

Nature Portfolio wishes to improve the reproducibility of the work that we publish. This form provides structure for consistency and transparency in reporting. For further information on Nature Portfolio policies, see our Editorial Policies and the Editorial Policy Checklist.

Statistics

For all statistical analyses, confirm that the following items are present in the figure legend, table legend, main text, or Methods section.

n/a	Confirmed	
☐	☒	The exact sample size (*n*) for each experimental group/condition, given as a discrete number and unit of measurement
☐	☒	A statement on whether measurements were taken from distinct samples or whether the same sample was measured repeatedly
☐	☒	The statistical test(s) used AND whether they are one- or two-sided<br>*Only common tests should be described solely by name; describe more complex techniques in the Methods section.*
☒	☐	A description of all covariates tested
☐	☒	A description of any assumptions or corrections, such as tests of normality and adjustment for multiple comparisons
☐	☒	A full description of the statistical parameters including central tendency (e.g. means) or other basic estimates (e.g. regression coefficient) AND variation (e.g. standard deviation) or associated estimates of uncertainty (e.g. confidence intervals)
☐	☒	For null hypothesis testing, the test statistic (e.g. F, t, r) with confidence intervals, effect sizes, degrees of freedom and P value noted<br>*Give P values as exact values whenever suitable.*
☒	☐	For Bayesian analysis, information on the choice of priors and Markov chain Monte Carlo settings
☒	☐	For hierarchical and complex designs, identification of the appropriate level for tests and full reporting of outcomes
☐	☒	Estimates of effect sizes (e.g. Cohen's *d*, Pearson's *r*), indicating how they were calculated

Our web collection on statistics for biologists contains articles on many of the points above.

Software and code

Policy information about availability of computer code

Data collection	BioTek Gen5 (v3.11), CFX Manager (v3.1), BD FACS Diva (8.0.1), ImageLab (6.0.1)
Data analysis	All statistical tests were done using GraphPrism7 (student’s two-sided t-test), FlowJo (V10.6.2), imageJ (v1.52a), MaxQuant (v1.5.1.2) or were part of the R package used for the analysis (MAGeCK-VISPR, DEP, CePa, msigdbr, gage). All computational analysis were performed using Python 3.7 (MAGeCK-VISPR v.0.5.6, TrimGalore! v.0.6.1, STAR v.2.7.9a, HTSeq v.1.99.2) or RStudio v.1.3.1093 (R v.4.0.3, biomaRt v.2.44.4, CePa v.0.7.0, clusterProfiler v.3.16.1, DEP v.1.10.0, gage v.2.38.3, msigdbr v.7.4.1, org.Hs.eg.db v.3.11.4, synergyfinder v.2.2.4, tidyverse v.1.3.1).

For manuscripts utilizing custom algorithms or software that are central to the research but not yet described in published literature, software must be made available to editors and reviewers. We strongly encourage code deposition in a community repository (e.g. GitHub). See the Nature Portfolio guidelines for submitting code & software for further information.

Data

Policy information about availability of data

All manuscripts must include a data availability statement. This statement should provide the following information, where applicable:

- Accession codes, unique identifiers, or web links for publicly available datasets
- A description of any restrictions on data availability
- For clinical datasets or third party data, please ensure that the statement adheres to our policy

Source data are provided as a Source Data file. The reference genome hg19 [https://www.ncbi.nlm.nih.gov/assembly/GCF_000001405.13/] is publicly available. The phosphoproteomics, CRISPR and RNA-seq data generated in this study have been deposited in the Zenodo database under accession code 10.5281/zenodo.6675783 [https://doi.org/10.5281/zenodo.6675783].

Field-specific reporting

Please select the one below that is the best fit for your research. If you are not sure, read the appropriate sections before making your selection.

☒ Life sciences ☐ Behavioural & social sciences ☐ Ecological, evolutionary & environmental sciences

For a reference copy of the document with all sections, see nature.com/documents/nr-reporting-summary-flat.pdf

Life sciences study design

All studies must disclose on these points even when the disclosure is negative.

Sample size

All in vitro experiments were performed in triplicates, except for the CRISPR screening. This was in line with the guidelines recommended by Carola A.S. Arndt for childhood and adolescent preclinical studies. For the CRISPR activation screening, no replicates were included as the library already had three independent sgRNAs. All candidate genes identified in the screening were validated in two cell lines using the same three sgRNA included in the library. For in vivo testing, sample size was decided based on previous experience with the models.

Data exclusions

No data was excluded

Replication

All replicates performed were succesful in reproducing the findings. The phosphoproteomics results were replicated in six different cellular models. The CRISPRa screen results were validated in two cellular models. The RNA-seq results were validated in two cellular models, using protein levels of proteins at different levels of the pathway.

Randomization

Mice harboring PDX tumor models were splitted in different groups. When possible, tumor size was equal in all mice. Groups were distributed aiming for a similar variability between groups at the beginning of the experiment.

Blinding

Blinding was no relevant to this study as the final measurements were based on objective measurements (e.g. tumor size, luminescence, fluorescence) and could not be possible to blind the researcher performing the experiments

Reporting for specific materials, systems and methods

We require information from authors about some types of materials, experimental systems and methods used in many studies. Here, indicate whether each material, system or method listed is relevant to your study. If you are not sure if a list item applies to your research, read the appropriate section before selecting a response.

Materials & experimental systems

n/a	Involved in the study	
☐	☒	Antibodies
☐	☒	Eukaryotic cell lines
☒	☐	Palaeontology and archaeology
☐	☒	Animals and other organisms
☐	☒	Human research participants
☒	☐	Clinical data
☒	☐	Dual use research of concern

Methods

n/a	Involved in the study	
☒	☐	ChIP-seq
☐	☒	Flow cytometry
☒	☐	MRI-based neuroimaging

Antibodies

Antibodies used

Described in Extended data Table 11

Validation

We used commercialy available antibodies, and followed the recommended dilutions. When possible, we chose antibodies referencing peer-reviewed articles where the application was well defined and the images showed high quality. All the references can be accessed through the corresponding antibody page from the manufacturer's website.

All antibodies obtained from CellSignaling Technology adhere to the Hallmarks of Antibody Validation (see https://www.cellsignal.com/about-us/cst-antibody-validation-principles). Antibodies are tested in models with known presence/absence of targt signal, signal strength is measured in cell lines representing a continuum of target expression levels, correlated with expression using antibody-independent assays, the signal is compared to the signal observed using antibodies targetting nonoverlapping epitopes of the target, evaluated in cell lines following heterologous expression of native target protein and validated using complementary assays. We confirmed that each antibody used met the quality control standards defined by Cell Signaling Technology, as per their own certificate of analysis. This includes antibodies #3700, #92356, #9664, #9422, #9427, #4695, #4370, #2251, #5281, #4499, #3377, #3873.

Santa Cruz Biotechnology provides an statement specifying application, dilution and species for which their antibodies have been tested. We maintained those conditions for all antibodies obtained from them, including #sc-374427, #sc-53993, #sc-98.

Thermo Fisher Scientific ensure the verification of the target specificity for antibody #MA5-14520. For antibodies #62-6520 and #G-21234, they confirm the specificity at the recommended applications and dilutions.

Merck Millipore provides a certificate of analysis for the antibodies #05-636 and #OP92-100UG. Bethyl laboratories provides a certificate of analysis for the antibody #A300-001A. Abcam ensures the specificity of the antibody #ab61065 in the assays tested (including WB, the application in this manuscript). Dianova provides an statement of the quality control of antibody #715-096-150.

Eukaryotic cell lines

Policy information about cell lines

Cell line source(s)	Rh41, Kym1 and Rh18 cells were a kind gift from Prof. Simone Fulda. Rh5, RMS and KFR were a kind gift from Prof. Beat Schäfer. Rh4, Rh30, T174, TE381.T, RD, C2C12, 5838, A4573, CHP, JR, SB, SK-N-MC, TC-71, HEK293T and CADO-ES1 were obtained from the American Type Culture Collection (ATCC, Manassas, Virginia).
Authentication	Cell line identity was verified by STR genotyping (Eurofins genomics, Luxenbourg)
Mycoplasma contamination	All cells tested negative for mycoplasma using MycoAlert (Lonza, Catalog number LT07-118)
Commonly misidentified lines (See ICLAC register)	SK-N-MC was correctly classified as Ewing's Sarcoma, instead of a neuroblastoma cell line (in accordance to the ICLAC register, which confirms SK-N-MC is an Ewing's Sarcoma cell line). Therefore, it's used is justified as part of our Ewing's Sarcoma cohort.

Animals and other organisms

Policy information about studies involving animals; ARRIVE guidelines recommended for reporting animal research

Laboratory animals	Mice strain: NOD.Cg-Prkdcscid Il2rgtm1Sug/JicTac (female between 6-8 week old) and NSG-H (NOD.Cg-Prkdcscid Hprtem1Mvw Il2rgtm1Wjl/MvwJ (6-8 week old, female and male)
Wild animals	No wild animals were used in this study
Field-collected samples	No field-collected samples were used in this study
Ethics oversight	All experiments were conducted according to the institutional animal protocols and the national laws and regulations and approved by the Charité University Medicine and MSKCC IACUC. Animals were IVC housed under sterile and standardized conditions (22°C +/- 1°C, 50% relative humidity, 12 hour light-dark cycle, autoclaved food, bedding material and tap water ad libitum).

Note that full information on the approval of the study protocol must also be provided in the manuscript.

Human research participants

Policy information about studies involving human research participants

Population characteristics	Volunteers were a 41F (Myo1), 32M (Myo2), 44F (Myo3), 52F (Myo4) and 21F (Myo5) who came to the hospital with a diagnosis (myalgia, cramps, myalgia, mialgya and family history of myopathy, respectively), but had no myopathology.
Recruitment	Volunteers were admitted for a muscle biopsy due to an existing diagnosis (myalgia, cramps, family history of myopathy) but no myopathology was diagnosed. We do not believe there is any implicit bias in our selection method.
Ethics oversight	All donors provided informed consent, and the myoblast isolation was done at the HELIOS Hospital Berlin Buch (Berlin, Germany) with the approval by the regulatory agencies (Ethics committee of Charité Universitätsmedizin Berlin, in compliance with the Declaration of Helsinki, approval number EA2/175/17).

Note that full information on the approval of the study protocol must also be provided in the manuscript.

Flow Cytometry

Plots

Confirm that:

☒ The axis labels state the marker and fluorochrome used (e.g. CD4-FITC).

☒ The axis scales are clearly visible. Include numbers along axes only for bottom left plot of group (a 'group' is an analysis of identical markers).

☒ All plots are contour plots with outliers or pseudocolor plots.

☒ A numerical value for number of cells or percentage (with statistics) is provided.

Methodology

Sample preparation	Described in methods
Instrument	BD LSR Fortessa
Software	BD FACS Diva for acquisition, FlowJo for analysis
Cell population abundance	In this study we used cell line models, and therefore no mix of cell types were used, and the population abundance was always 100%
Gating strategy	First the cell population was isolated using SSC-A vs FSC-A, to exclude debris form further analysis. Then, single cells were selected using FSC-A vs FSC-H. Finally, the staining of interest was selected and gated according to the specific experiment. An exemplary of each gating strategy is shown in Extended data Fig. 2

☒ Tick this box to confirm that a figure exemplifying the gating strategy is provided in the Supplementary Information.
